# HDAC8-mediated inhibition of EP300 drives a transcriptional state that increases melanoma brain metastasis

**DOI:** 10.1038/s41467-023-43519-1

**Published:** 2023-11-29

**Authors:** Michael F. Emmons, Richard L. Bennett, Alberto Riva, Kanchan Gupta, Larissa Anastasio Da Costa Carvalho, Chao Zhang, Robert Macaulay, Daphne Dupéré-Richér, Bin Fang, Edward Seto, John M. Koomen, Jiannong Li, Y. Ann Chen, Peter A. Forsyth, Jonathan D. Licht, Keiran S. M. Smalley

**Affiliations:** 1 Department of Tumor Biology, Moffitt Cancer Center, 12902 Magnolia Drive, Tampa, FL 33612 USA; 2 UF Health Cancer Center, 2033 Mowry Road, University of Florida, Gainesville, FL 32610 USA; 3 Bioinformatics Core, Interdisciplinary Center for Biotechnology Research, University of Florida, 2033 Mowry Road, Gainesville, FL 32610 USA; 4 Department of Neuro-Oncology, Moffitt Cancer Center, 12902 Magnolia Drive, Tampa, FL 33612 USA; 5 Proteomics & Metabolomics Core, Moffitt Cancer Center, 12902 Magnolia Drive, Tampa, FL 33612 USA; 6 Department of Biochemistry & Molecular Medicine, School of Medicine & Health Sciences, George Washington Cancer Center, George Washington University, 2300 Eye Street, Washington, DC 20037 USA; 7 Department of Molecular Oncology, Moffitt Cancer Center, 12902 Magnolia Drive, Tampa, FL 33612 USA; 8 Department of Bioinformatics and Biostatistics, Moffitt Cancer Center, 12902 Magnolia Drive, Tampa, FL 33612 USA; 9 Department of Cutaneous Oncology, Moffitt Cancer Center, 12902 Magnolia Drive, Tampa, FL 33612 USA

**Keywords:** Melanoma, Epigenetics, Cancer genomics, Metastasis, Cancer epigenetics

## Abstract

Melanomas can adopt multiple transcriptional states. Little is known about the epigenetic drivers of these cell states, limiting our ability to regulate melanoma heterogeneity. Here, we identify stress-induced HDAC8 activity as driving melanoma brain metastasis development. Exposure of melanocytes and melanoma cells to multiple stresses increases HDAC8 activation leading to a neural crest-stem cell transcriptional state and an amoeboid, invasive phenotype that increases seeding to the brain. Using ATAC-Seq and ChIP-Seq we show that increased HDAC8 activity alters chromatin structure by increasing H3K27ac and enhancing accessibility at c-Jun binding sites. Functionally, HDAC8 deacetylates the histone acetyltransferase EP300, causing its enzymatic inactivation. This, in turn, increases binding of EP300 to Jun-transcriptional sites and decreases binding to MITF-transcriptional sites. Inhibition of EP300 increases melanoma cell invasion, resistance to stress and increases melanoma brain metastasis development. HDAC8 is identified as a mediator of transcriptional co-factor inactivation and chromatin accessibility that drives brain metastasis.

## Abstract (web summary)

The drivers of melanoma brain metastases (MBM) remain poorly understood. Here, the authors identify stress-induced HDAC8 activity as the driver of a neural crest-stem cell like transcriptional state that leads to MBM, and explore the molecular mechanism that drives this transition.

## Introduction

Cutaneous melanoma is the deadliest form of skin cancer, with a propensity to aggressively metastasize to multiple organs^1^. One common site of melanoma metastasis development is the brain, with 40-60% of patients with advanced melanoma showing evidence of CNS involvement^2,3^. Left untreated, melanoma brain metastases (MBMs) progress rapidly, with most patients dying within 3 months^4^. Little is known about the molecular drivers of MBM development. Most studies to date have focused upon the role of phosphatase and tensin homolog (PTEN) loss and hyperactivation of AKT signaling as potential drivers of MBM development^5,6^. There is evidence that clinical specimens of MBM have reduced PTEN expression/increased AKT phosphorylation compared to melanoma metastases in other sites^7^. Additionally, studies in mouse models have shown that myristolated AKT1 on a background of PTEN loss increased the formation of MBM^8^. Although there is evidence from lung and breast cancers that brain metastasis development may be associated with the acquisition of secondary genetic mutations (e.g. ERBB2 and components of the PI3K/AKT/mTOR pathway)^9^, MBMs tend to have similar mutational landscapes to extracranial tumors^10^, suggesting a possible role for epigenetic drivers.

Melanoma is a heterogeneous tumor whose constituent cancer cells adopt a range of transcriptional states. High dimensional single cell analysis has identified up to 4 melanoma cell states that are defined as (1) a de-differentiated/invasive cell state (marked by low expression of the melanocyte-lineage factor MITF, high Axl expression and invasion/adhesion genes), (2) a neural crest stem cell (NCSC)-like state (characterized by low MITF expression and increased neural-crest gene expression), (3) a melanocytic/differentiated state (e.g. expressing melanocyte lineage genes such as MITF and those involved in pigmentation), and (4) a transitory state with a gene expression profile reflecting both the neural-crest and differentiated phenotypes^11,12^. The presence of these cell states in both melanoma cell lines and patient specimens has been confirmed by multiple studies^13–16^. Treatment of melanomas with targeted therapy alters the heterogeneity of the tumor leading to an expansion of melanoma cell states that are more drug resistant, de-differentiated and more invasive^17–19^. How these transcriptional states are regulated and how these individually contribute to tumor progression remains unclear.

We previously identified the class I histone deacetylase HDAC8 as a driver of BRAF and BRAF-MEK inhibitor resistance in *BRAF*-mutant melanoma^20^. As HDAC8 activity also increased in response to other stresses, we reasoned that increased HDAC8 activity could be critical for melanoma transcriptional state switching.

Here, we show that HDAC8 is a stress-induced regulator of transcriptional state changes in melanoma cells. Phenotypically, HDAC8 drives the melanoma cells to adopt an amoeboid phenotype that promotes melanoma cell survival under shear stress conditions, enhances invasive capacity and increases the formation of brain metastases. Mechanistically, HDAC8 activation (1) deacetylates EP300, inhibiting its enzymatic activity, switching its association from MITF sites to Jun sites and (2) increases H3K27 acetylation and chromatin accessibility at Jun-target gene transcriptional sites. Our work provides evidence that stress-induced HDAC8 activity regulates an invasive melanoma cell state that increases melanoma brain metastasis development.

## Results

### HDAC8 activity is increased following exposure of melanoma cells to stress

**Fig. 1:**
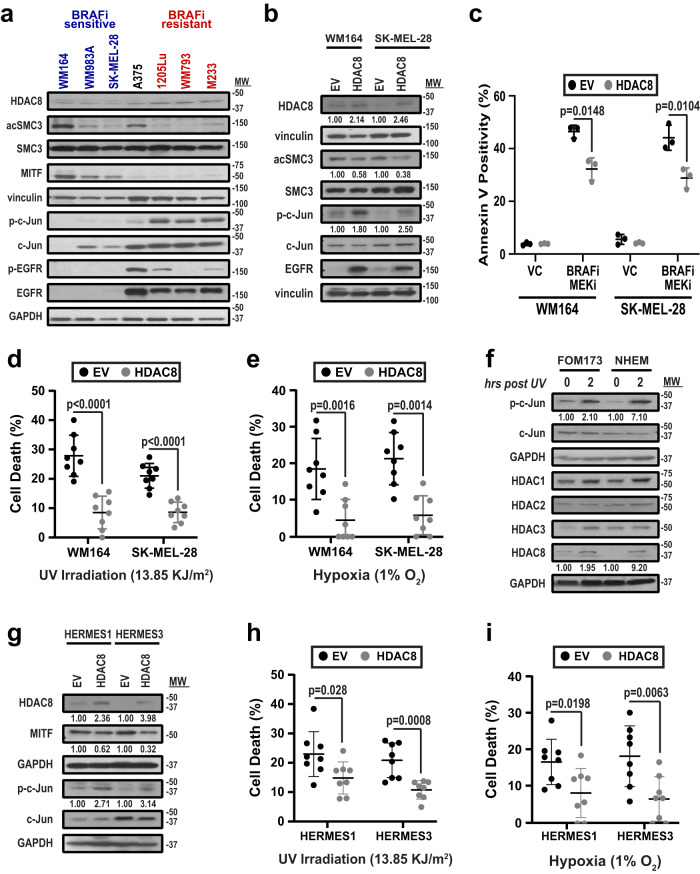
HDAC8 expression confers stress resistance in melanocytes and melanoma. **a** Cell lines treated with vemurafenib (BRAFi) were organized into BRAFi sensitive (IC50 < 1 µM) BRAFi intermediate (IC50 = 1), and BRAFi resistant (IC50 > 1 µM). Cells were probed for HDAC8, acetylated SMC3 (acSMC3), SMC3, phospho-EGFR (p-EGFR), EGFR, phospho-c-Jun (p-c-Jun), c-Jun, and MITF by immunoblot. **b** Cells transfected with an empty vector (EV) or HDAC8 construct were probed for HDAC8, acSMC3, SMC3, p-c-Jun, c-Jun, and EGFR by immunoblot with ImageJ quantification. **c** Cells were treated with vehicle (1:1000 DMSO, VC), or with BRAF and MEK inhibition (100 nmol/L dabrafenib, 10 nmol/L trametinib, BRAFi-MEKi) for 72 h. Apoptosis was measured by Annexin V APC staining. Significance was determined by a one-way ANOVA followed by a 2-tailed *t* test with *=*p* < 0.05 (WM164: *p* = 0.0148 and SK-MEL-28: *p* = 0.0104). Cells were treated with (**d**) 13.85 KJ/m^2^ UV irradiation or (**e**) 1% O_2_ for 24 h followed by cell death measurement by trypan exclusion. Significance was determined by a one-way ANOVA followed by a 2-tailed *t* test with ***=*p* < 0.001 and **=*p* < 0.01. In (**d**), WM164 *p* value < 0.0001 and SK-MEL-28 *p* value < 0.0001. In (**e**), WM164 *p* value = 0.0016 and SK-MEL-28 *p* value = 0.0014. **f** Primary melanocyte cells were treated with 13.85 KJ/m^2^ UV irradiation for indicated time points. Lysates were collected and probed for p-c-Jun, c-Jun, HDAC1, HDAC2, HDAC3, and HDAC8 by immunoblot with ImageJ quantification. **g** HERMES1 and 3 cells (melanocytes) were transfected with an EV or HDAC8 construct and probed for HDAC8, MITF, p-c-Jun, and c-Jun by immunoblot with ImageJ quantification. HERMES1 and 3 cells were treated with (**h**) 13.85 KJ/m^2^ UV irradiation or (**i**) 1% O_2_ for 24 h followed by cell death measurement by trypan exclusion. Significance was determined by a one-way ANOVA followed by a 2-tailed *t* test with *=*p* < 0.05 and **=*p* < 0.01, and ***=*p* < 0.001. In (**h**), HERMES1 *p* value = 0.028 and HERMES3 *p* value = 0.0008. In (**i**), HERMES1 *p* value = 0.0198 and HERMES3 *p* value = 0.0063. All experiments were run 3 independent times with an n of 3 in each cohort in (**c**) and an n of 8 in each cohort in (**d**), (**e**), (**h**) and (**i**). All data are presented as mean values ±SD. Source data are provided as a Source Data file.

We began by determining if HDAC8 was an epigenetic driver of cell state changes in melanoma. Immunoblot profiling of a melanoma cell line panel demonstrated that higher expression of the melanocyte lineage transcription factor MITF correlated with lower HDAC8 expression and reduced levels of signaling molecules known to be regulated by HDAC8 including acetylated SMC3, EGFR (both total and phospho) and c-Jun (both total and phospho)^20,21^ (Fig. 1a). The cell lines were ranked according to their baseline sensitivity to the BRAF inhibitor (BRAFi) vemurafenib^18^ and demonstrated a link between higher MITF expression, lower HDAC8 expression and increased BRAFi sensitivity (Fig. 1a). Likewise, introduction of HDAC8 into melanoma cell lines with low endogenous HDAC8 expression increased phospho-Jun and EGFR levels (Fig. 1b).

As transcriptional switching could be a possible response to exogenous stress, we next asked whether introduction of HDAC8 increased the survival of melanocytic cells under different microenvironmental conditions. Increased HDAC8 expression led to increased survival of melanoma cells exposed to hypoxia, UV irradiation and after treatment with BRAF-MEKi therapy (Fig. 1c–e and Supplementary Fig. 1a, b). Stable expression of HDAC8 in a BRAFi sensitive melanoma cell line significantly shifted the BRAFi vemurafenib IC_50_ and inhibited the proliferation rate (Supplementary Fig. 1c, d). To determine if this was a conserved response between melanocytes and melanoma cells, we treated 2 primary human melanocyte cell lines (FOM173 and NHEM) with UV irradiation and noted increased expression of HDAC8 and increased phosphorylated c-Jun expression (Fig. 1f). Some minor changes in other HDACs, such as HDAC1 were also noted. Similar HDAC8 responses were also seen in human melanoma cells treated with either UV irradiation of hypoxia (Supplementary Fig. 1e). Expression of HDAC8 in two further human melanocyte lines (HERMES1 and HERMES3) led to decreased expression of MITF, and increased levels of phospho-c-JUN (Fig. 1g), and conferred increased survival when the cells were subjected to either hypoxia or UV irradiation (Fig. 1h, i).

### HDAC8 drives a transcriptional switch in melanoma cells

**Fig. 2:**
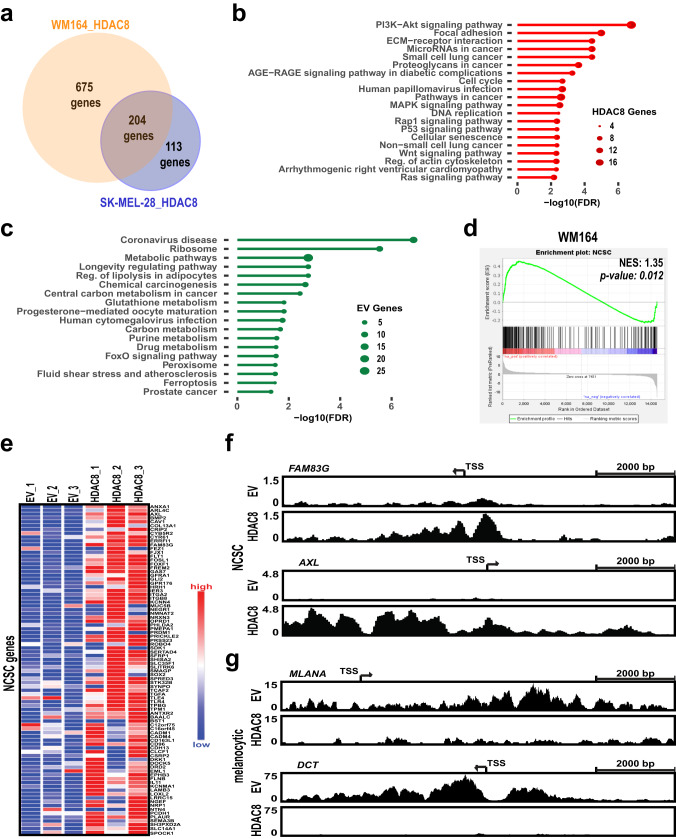
HDAC8 drives a transcriptional switch in melanoma cells. **a** RNA-seq was performed in triplicate on EV and HDAC8 expressing WM164 and SK-MEL-28 cells with significantly changed genes assigned a log_2_ fold change value ±0.58 and a 2 sided *p* value < 0.05 without multiple hypothesis testing. The number of uniquely and overlapping upregulated genes upon HDAC8 expression are shown in the Venn diagram. **b**, **c** A KEGG pathway analysis was performed on overlapping significantly changed genes using RNA-seq data for both WM164 (EV vs HDAC8) and SK-MEL-28 (EV vs HDAC8) expressing cells. Pathways enhanced in HDAC8 expressing cells are shown in (**b**) and pathways downregulated in HDAC8 expressing cells are shown in (**c**). **d** A Ranked Order analysis was performed on NCSC and NCSC/undifferentiated genes using GSEA software. Shown is the normalized enrichment score (NES) and 2-sided nominal *p* value of the dataset without multiple hypothesis testing. Significance is determined by having a nominal *p* value < 0.05. **e** Heat maps were made of significantly altered NCSC genes comparing EV and HDAC8 expressing cells. **f**, **g** ChIP-Seq was performed on EV and HDAC8 expressing WM164 cells using an acetyl-H3K27 antibody followed by interrogation of gene promoter regions. HDAC8 expression increased H3K27 acetylation in promoter regions in (**f**) NCSC genes (*FAM83G* and *AXL*) and decreased H3K27 acetylation in promoter regions in (**g**) melanocytic genes (*MLANA* and *DCT*).

RNA-seq analyses were performed on two melanoma cell lines (WM164 and SK-MEL-28) to determine how upregulation of HDAC8 reshaped the transcriptional landscape (Supplementary Fig. 2a, b). Integration of the cell lines revealed that 204 common genes were significantly increased and 163 common genes were significantly decreased with HDAC8 expression (Fig. 2a and Supplementary Fig. 2c). A Kyoto Encyclopedia of Genes and Genomics (KEGG) pathway analysis revealed that HDAC8 expression increased enrichment for pathways important for melanoma migration including PI3K-AKT signaling, Focal Adhesion, ECM-Receptor Interactions, Regulation of Actin Cytoskeleton, MAPK signaling and RAP1 signaling (Fig. 2b) while decreasing enrichment of some Metabolic Pathways, including glutathione and purine metabolism (Fig. 2c). As these data suggested the HDAC8-driven transcriptional program was associated with an invasive, metastatic phenotype we next determined whether increased HDAC8 expression also correlated with previously identified melanoma transcriptional states. Recent high-dimensional analyses of melanoma heterogeneity^11^, since confirmed by multiple additional studies^13–16^, identified at least 4 different cell states, including an undifferentiated embryonic stem cell (ESC) state, a neural crest stem cell (NCSC)-like state, a transitory state, and a differentiated melanocyte state^11^. Analysis of our dataset demonstrated that HDAC8 expression enriched for genes associated with the NCSC and the transitory state in SK-MEL-28 cells and the NCSC and undifferentiated state in the WM164 cells (Fig. 2d, e, Supplementary Fig. 2d, e).

We next verified increased initiation of transcriptional activity of NCSC genes by analyzing H3K27ac ChIP-Seq data in the promoter regions of NCSC genes in WM164 and 1205Lu cells. Genes associated with the NCSC state, including *AXL, FAM83G, FOSL1*, and *SOX2* (see Supplementary Data 1 for full gene list) had increased H3K27ac acetylation in HDAC8 expressing cells (Fig. 2f, Supplementary Fig. 3a, b) while genes associated with a melanocytic state, including *MLANA, DCT, TYR*, and *PMEL* had decreased H3K27ac acetylation in HDAC8 expressing cells (Fig. 2g, Supplementary Fig. 3a, c).

### The HDAC8-driven transcriptional state leads to an invasive amoeboid phenotype

**Fig. 3:**
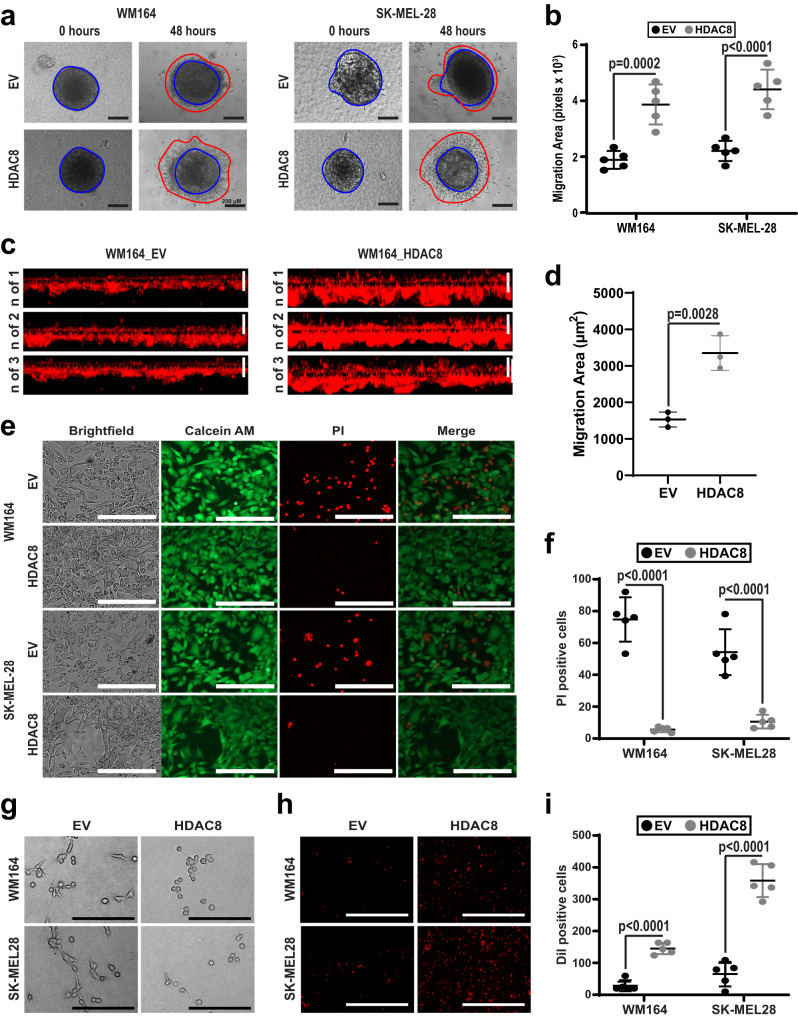
HDAC8 increases invasion and an amoeboid-like state in melanoma cells. **a** HDAC8 expression increases invasion in a collagen-implanted spheroid assay. EV and HDAC8 expressing melanoma spheroids were plated in collagen for indicated time points. Scale bars = 500 µm. **b** Spheroid invasion area was quantified using ImageJ software. Significance was determined using a 2-tailed student’s *t* test with ***p < 0.001. (WM164: *p* = 0.0002 and SK-MEL-28: *p* < 0.0001). **c** HDAC8 expression increases invasion into Matrigel. EV and HDAC8 expressing cells were overlayed onto transwell inserts coated with Matrigel and allowed to invade for 48 h followed by imaging by confocal microscopy. Scale bars = 100 µm. **d** Invasion area was quantified using ImageJ software. Significance was determined using a 2-tailed student’s *t* test with **p < 0.01. (*p* = 0.0028). **e** HDAC8 increases melanoma survival in shear stress/flow assays. Cells were incubated for 24 h under continual shear stress conditions of 10 dyne/cm2. Cells were stained with Calcein AM/PI and imaged for cell viability. Scale bars = 250 µm. **f** Data shows numbers of dead cells (PI-positive cells) per image from (**e**). Significance was determined using a 2-tailed student’s *t* test with ***p < 0.001. (WM164: *p* < 0.0001 and SK-MEL-28: *p* < 0.0001). **g** HDAC8 leads to the adoption of an amoeboid-like morphology in melanoma cells. HDAC8 and EV expressing cells were plated on collagen overnight followed by brightfield image collection. Scale bars = 250 µm. **h** HDAC8 enhances transendothelial cell migration. DiI-stained HDAC8 and EV cells were allowed to migrate through a HUVEC coated transwell membrane for 1 h followed by imaging with fluorescence microscopy. Scale bars = 1000 µm. **i** Quantification of the DiI-stained cells that had migrated through the endothelial cell monolayer (cells per image). Significance was determined using a 2-tailed student’s *t* test with ***p < 0.001. (WM164: *p* < 0.0001 and SK-MEL-28: *p* < 0.0001). Experiments were run 3 independent times with an n of 3 in (**d**) and an n of 5 in (**b**), (**f**), and (**i**). All data are presented as mean values ± SD. Source data are provided as a Source Data file.

Cells that metastasize to the brain must overcome multiple obstacles including survival under the high shear stress conditions of the circulatory system, extravasation through the blood-brain-barrier and growth in the protease-rich environment of the brain parenchyma^22–24^. As many genes that were upregulated in our RNA-seq and ChIP-Seq analysis are linked to increased invasion, we performed 3D spheroid collagen invasion and Matrigel invasion assays and found that expression of HDAC8 led to increased WM164 and SK-MEL-28 cell invasion when compared to EV cells (Fig. 3a–d).We next asked whether HDAC8 increased the resilience of melanoma cells to shear stress through use of a flow chamber system that mimics the levels of shear experienced in the general circulation^25,26^. Exposure of WM164 and SK-MEL-28 melanoma cells to high levels of fluid shear stress (10 dyne/cm2) over 24 h led to high levels of cell death in the control (EV) cells that were reduced by 80–90% in HDAC8-expressing melanoma cells (Fig. 3e, f). Many of the genes upregulated by HDAC8 were involved in cytoskeletal rearrangement, including *SOX2* and *PROM1* that are associated with an amoeboid phenotype^27^. In line with this observation, HDAC8 expressing WM164 and SK-MEL-28 melanoma cells adopted a rounded, amoeboid phenotype after being plated onto collagen (Fig. 3g). As the amoeboid phenotype can allow cells to squeeze through tight spaces, such as those associated with exiting blood vessels and crossing the blood brain barrier, we performed transendothelial cell migration assays and found that HDAC8 expression led to a ten-fold increase in transmigration of melanoma cells through confluent endothelial cell layers (Fig. 3h, i). Collectively, these findings suggested that the HDAC8-driven transcriptional program could increase invasion of melanoma cells in vivo. To test this, we injected either GFP-labeled HDAC8 expressing or control EV WM164 cells into the tail veins of mice and collected the lungs after 10 or 24 h. It was found that HDAC8-expression led to an increase in lung colonization that was significantly different from control cells at 10hrs (Supplementary Fig. 4a, b), but was no longer significant by 24 h (Supplementary Fig. 4c, d).

### Introduction of HDAC8 increases the development of brain metastases in mice

**Fig. 4:**
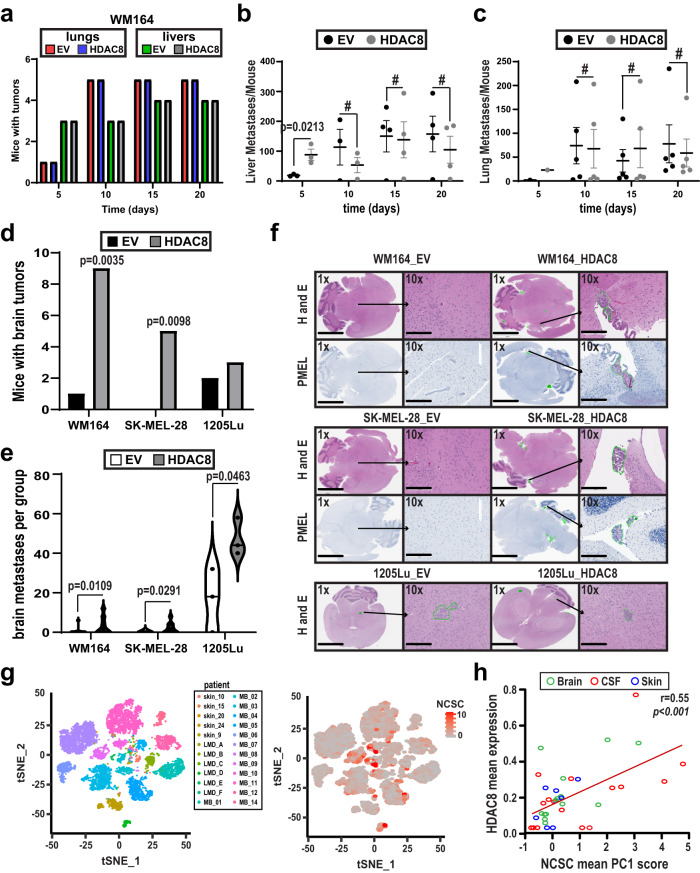
HDAC8 increases the establishment of melanoma brain metastases. **a**–**c** HDAC8 and EV expressing WM164 cells were introduced into NOD.CB17-Prkdcscid/J mice by intracardiac injection. **a** Tumors were allowed to establish for indicated time points with numbers of metastases measured in H&E sections of the liver and lung. Number of metastases present in the (**b**) liver and (**c**) lungs were calculated using Imagescope. Significance in (**b**) and (**c**) was determined by a one-way ANOVA followed by a 2-tailed post hoc *t* test with *=*p* < 0.05, and #=*p* > 0.05. In (**b**), day 5 *p* = .0213. Data are presented as mean values of 5 mice ±SD. **d**–**f** HDAC8 and EV expressing WM164, SK-MEL-28 and 1205Lu cells were introduced into NOD.CB17-Prkdcscid/J mice by intracardiac injection and allowed to incubate for 14 days. **d** Number of mice with brain tumors were counted for each condition using Imagescope software. Significance in (**d**) was determined by a 2-sided chi-squared test on an n of 20 mice in WM164 cells and an n of 10 mice in SK-MEL-28 cells with **=*p* < 0.01. (WM164: *p* = 0.0035 and SK-MEL-28: *p* = 0.0098). **e** Number of brain metastases per mouse brain under each condition were counted using Imagescope software. Significance was determined by a one-way ANOVA followed by a 2-tailed *t* test on an n of 20 mice in WM164 cells, an n of 10 mice in SK-MEL-28 cells and an n of 3 mice in 1205Lu cells with *=*p* < 0.05 (WM164 *p* = 0.0109, SK-MEL-28 *p* = 0.0291, 1205Lu *p* = 0.0463). **f** Embedded brain sections were stained with H&E and an IHC for PMEL (gp100) to determine tumor formation. Scale bars in 1x images = 4 mm. Scale bars in 10x images = 300 µm. **g** Patient-derived scRNA-Seq data (#’s=melanoma cutaneous samples, LMD melanoma leptomeningeal metastasis samples, MB melanoma brain metastases samples) were interrogated for expression of NCSC genes. (Left: sample level data for melanoma cells. Right: Expression of the NCSC gene signature in the melanoma cells). **h** A Spearman correlation analysis was run to determine the relationship of HDAC8 expression and the NCSC gene expression signature from the human scRNA-Seq data. A *p* value *p* < 0.05 corresponds to a significant correlation. Source data are provided as a Source Data file.

We next asked whether the HDAC8-driven transcriptional state altered the patterns of metastatic seeding in vivo. Control or HDAC8 expressing WM164 melanoma cells were injected into the left ventricle of the hearts of mice and organs collected at 5 day intervals to identify potential metastatic sites. There was little difference in the total incidence of liver or lung metastases between the HDAC8 expressing and EV group (counted as >1 metastasis per mouse at each site) (Fig. 4a). The number of liver metastases was significantly higher in the HDAC8 expressing cohort, compared to the EV cells at day 5, but this was no longer significant at subsequent time points (Fig. 4b, images in Supplementary Fig. 5a). Analysis of lungs revealed similar dynamics of metastasis development between the HDAC8 expressing and EV control over time (Fig. 4c, images in Supplementary Fig. 5b). There was no significant differences in the areas of the lung and liver metastases between the EV and HDAC8 expressing cells (Supplementary Fig. 6a, b). Analysis of other organs, including the ovaries, heart and spleen did not show any differences in metastatic development between HDAC8 overexpressing and EV control groups (Supplementary Fig. 6c, d). The weight of the EV and HDAC8 mice were similar at endpoint suggesting there were no general adverse effects on the mouse health between the two experimental groups (Supplementary Fig. 6e).

We next focused on the propensity for HDAC8 expressing melanoma cells to form melanoma brain metastases. Following intracardiac injection, HDAC8 expressing SK-MEL-28, WM164 and 1205Lu melanoma cells resulted in significantly more brain metastases compared to the control (EV) cells (Fig. 4d–f: Supplementary Figs. 7, 8). Sectioning of the whole brains (at 150 um intervals) from mice injected via the intracardiac route with either EV or HDAC8-expressing 1205Lu confirmed the HDAC8-associated increase in brain metastasis formation (Supplementary Fig. 7, Supplementary Fig. 8c). Neuropathologic examination (by study pathologist, RM) identified tumors involving the ependymal surfaces of the temporal horns of the lateral ventricles, as well as invasion into the brain parenchyma, with the tumor border encroaching on the perivascular spaces. Staining of the brains for blood vessel markers (anti-CD31) confirmed that the melanoma cells had extravasated, with evidence of angiogenesis in the lesions (Supplementary Fig. 8a–c).

To address the clinical relevance of these findings we interrogated our recent single cell RNA-seq analysis of clinical melanoma samples^28,29^. These analyses showed a significant correlation between HDAC8 expression and the NCSC-like gene expression profile in the human melanoma cells (Fig. 4g, h). A more detailed breakdown by site of metastasis demonstrated a significant association between HDAC8 expression and the NCSC-like state for the LMD and brain metastasis samples, but not the skin metastases. However, this was likely due to the low numbers of tumor cells in the skin metastasis samples. Melanoma brain metastases are often enriched for an OXPHOS metabolism gene expression signature^10^. To investigate this, we interrogated the human melanoma scRNA-seq data and found that HDAC8 expression was associated with an OXPHOS gene signature (Supplementary Fig. 9a). Mechanistic support for HDAC8 driving this metabolic phenotype was demonstrated through Seahorse XF Cell Mito Stress analyses which showed that HDAC8 expression led to an increased rate of mitochondrial respiration (OXPHOS) (Supplementary Fig. 9b, c).

### HDAC8 increases chromatin accessibility and H3K27ac of Jun-targeted genes

**Fig. 5:**
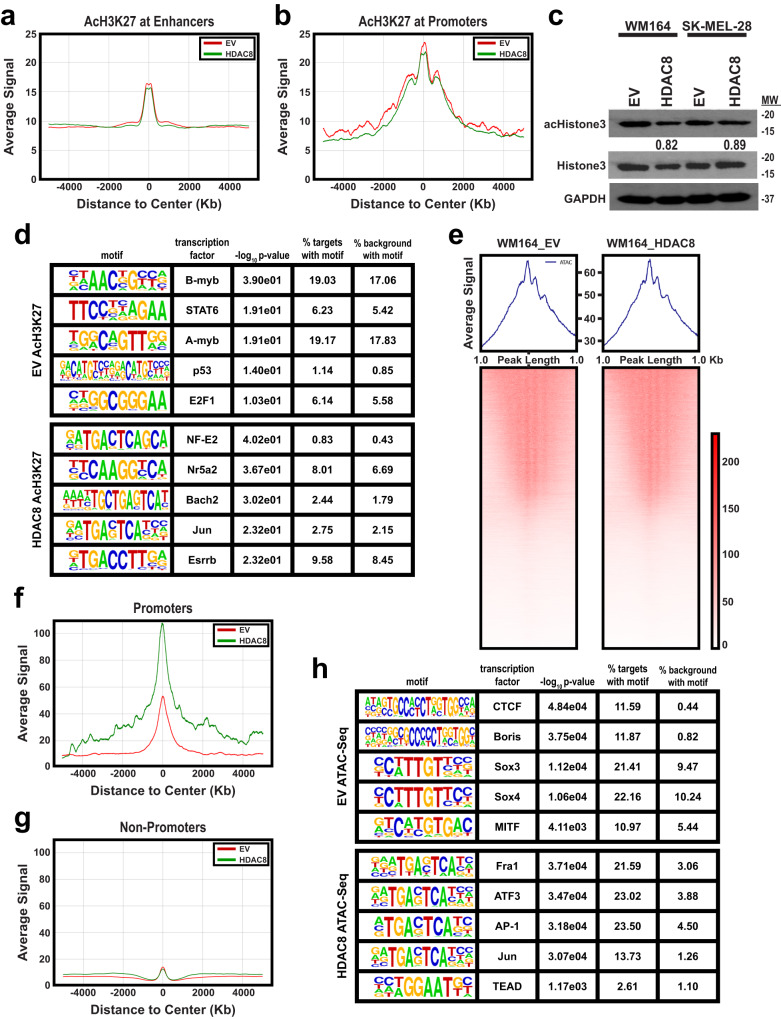
HDAC8 activation induces changes in chromatin accessibility of Jun and MITF targeted genes. A ChIP-Seq analysis was performed on HDAC8 expressing and EV expressing melanoma cells at both (**a**) promoter and (**b**) enhancer regions. **c** Cell lines were probed for acetyl-Histone3 and total-Histone 3 levels by immunoblot. Levels of acetyl-Histone3 were normalized to total-Histone3 and quantified by ImageJ. **d** A HOMER analysis was performed on HDAC8 and EV expressing cells. Shown are enriched transcription factor binding motifs in HDAC8 and EV expressing cells. **e**–**g** ATAC-Seq was performed on HDAC8 expressing and EV expressing cells. **e** A global analysis of all open chromatin was performed. A global analysis was performed on HDAC8 expressing and EV expressing melanoma cells at both (**f**) promoter and (**g**) non-promoter regions. **h** HDAC8 expression increases accessibility in Jun targeted genes and decreases accessibility of MITF targeted genes. A HOMER analysis was performed on ATAC-Seq data. HDAC8 was associated with increased accessibility at Jun binding sites while the EV control had increased accessibility at MITF binding sites. Source data are provided as a Source Data file.

We next used ATAC-Seq and ChIP-Seq to determine how HDAC8 regulated transcriptional reprogramming. Global analysis of ChIP-Seq data revealed HDAC8 expression to have little global effect on H3K27ac at either promoter or enhancer regions (Fig. 5a, b), there was likewise little alteration of global acetyl-H3 histone protein levels (Fig. 5c). A HOMER analysis of H3K27ac ChIP-Seq data revealed an increase in H3K27 acetylation at Jun sites when HDAC8 was introduced, whereas control cells exhibited an increase in H3K27 acetylation at transcription factors important for cell cycle progression, including B-myb and A-myb (Fig. 5d). An ATAC-seq analysis of peak numbers/length in genes showed similar levels of accessibility and DNA fragment sizes in HDAC8 expressing cells and EV cells (Fig. 5e). While overall accessibility was similar throughout the genome, promoters showed increased accessibility in HDAC8 expressing cells (Fig. 5f) whereas little difference was seen at non-promoter sites (Fig. 5g). An analysis of the motifs with increased accessibility upon HDAC8 expression included multiple AP-1 transcription factors (Fra1, ATF3, Jun) and TEADs (Fig. 5h, Supplementary Fig. 10a, b). Multiple motifs also showed downregulation upon HDAC8 introduction including CTCF, multiple SOX transcription factors, and the melanocyte lineage transcription factor MITF (Fig. 5h, Supplementary Fig. 10a, b). In support of our ChIP-Seq data showing few changes in global H3K27ac histone acetylation, additional immunoblot analyses did not identify any consistent changes in total H3K9, H4K16 or H4K20 acetylation upon introduction of HDAC8 (Supplementary Fig. 10c).

### HDAC8 inactivates EP300 leading to increased Jun transcriptional activity

**Fig. 6:**
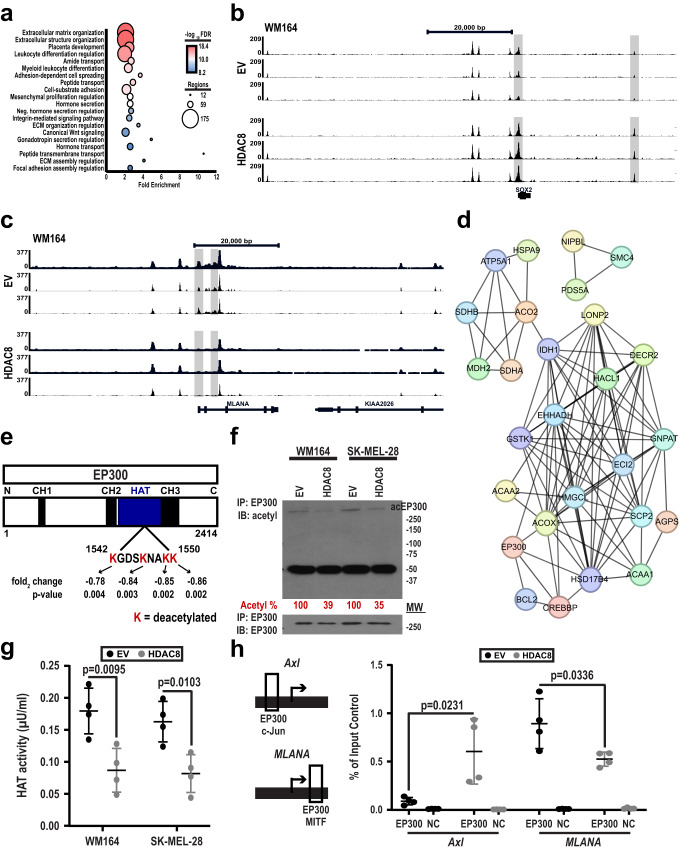
HDAC8 deacetylates and inactivates EP300 leading to increased JUN transcriptional activity. **a** A GREAT analysis was performed on genes with enhanced accessibility/H3K27 acetylation around their transcription start sites. **b**, **c** ATAC-seq analysis of EV and HDAC8 expressing WM164 melanoma cells. There is increased accessibility in the promoter region of the (**b**) NCSC gene SOX2 while there is decreased accessibility in the promoter region of the (**c**) melanocyte gene MLANA upon HDAC8 expression. Data show 3 independent tracks. **d**, **e** A mass spectrometry-based acetylomic analysis was performed on HDAC8 and EV expressing WM164 cells. **d** Proteins deacetylated by HDAC8 identified by mass spectrometry were organized into signaling hubs using STRING. **e** Mass spectrometry was used to identify significantly changed lysine acetylation sites in the HAT domain of EP300 are shown. Figures shows the 4 identified lysine residues deacetylated upon introduction of HDAC8. Significance was determined using a 2-tailed student’s *t* test with *p < 0.05. **f** HDAC8 deacetylates EP300. Immunoprecipitation assays were performed with EP300 in the cell lines indicated. Lysates were probed for acetyl-lysine levels by immunoblot. Levels of acetyl-EP300 were normalized to EP300 input controls and quantified by ImageJ. **g** HDAC8 inhibits the HAT activity of EP300. Immunoprecipitation assays were performed with EP300 in two independent melanoma cell lines (WM164 and SK-MEL-28). Collected lysates were analyzed for EP300 mediated H4 histone acetylation. Significance was determined by a 2-tailed student’s *t* test with *=*p* < 0.05 and **=*p* < 0.01 (WM164: *p* = 0.0095 and SK-MEL-28: *p* = 0.0103). **h** HDAC8 expression increases the binding of EP300 to Jun promoters and decreases binding to MITF promoters. A ChIP assay was performed on EP300 binding to the promoter region of *Axl* and *MLANA*. Significance was determined by a one-way ANOVA followed by a post hoc 2-tailed *t* test with *=*p* < 0.05 (WM164: *p* = 0.0231 and SK-MEL-28: *p* = 0.0336). Experiments were run 3 independent times with an n of 4 in each cohort. All data are presented as mean values ±SD. Source data are provided as a Source Data file.

Bioinformatic integration of ATAC-Seq and ChIP-Seq data suggested HDAC8 expression confers enhancement of accessibility/transcriptional activation of genes in pathways associated with extracellular matrix assembly, cell spreading and differentiation (Fig. 6a, Supplementary Fig. 11a). Although the ATAC-Seq and ChIP-Seq data showed HDAC8 mediated few global changes in H3K27ac or chromatin accessibility, changes at discrete Jun and MITF sites were noted in both the WM164 and 1205Lu cell lines. Interestingly, many of the genes implicated in the NCSC phenotype, such as *SOX2* are regulated by c-Jun and demonstrated increased accessibility/H3K27 acetylation in the HDAC8 expressing cells (Fig. 6b, Supplementary Fig. 11b), while MITF regulated genes, such as *MLANA* (involved in the melanocytic phenotype) had decreased accessibility (Fig. 6c, Supplementary Fig. 11c). Of relevance to the development of MBM, we noted that HDAC8 expression led to increased gene expression, H3K27ac and chromatin accessibility of multiple Serpins (e.g., Serpin E1, E2 and A1), protease inhibitors that facilitate the establishment of brain metastases^23^ (Supplementary Fig. 12a–c). Together these data suggested that HDAC8 introduction led to a switch from MITF-driven transcriptional programs to those regulated by c-Jun.

As the effects of HDAC8 on global chromatin accessibility were relatively minor we next asked whether HDAC8 mediated any of its effects through regulation of non-histone targets. A proteomics analysis was performed to determine how HDAC8 affected the “acetylome” of melanoma cells (Supplementary Fig. 13a shows the workflow). Significantly deacetylated proteins involved in the regulation of lipid metabolism were identified STRING analysis (Fig. 6d: acetylated peptides are shown in Supplementary Data 2). One of the central hubs identified was the histone acetyltransferase (HAT) EP300/CREBBP, which is known to acetylate multiple proteins including MITF and c-Jun^30,31^. Introduction of HDAC8 had minimal effects upon EP300 expression (Supplementary Fig. 13b). An analysis of the structure of EP300 identified 4 lysine residues in the HAT domain of EP300 (Lysine’s 1542, 1546, 1549, and 1550) that were deacetylated upon expression of HDAC8 (Fig. 6e). The ability of HDAC8 to deacetylate EP300 was confirmed by immunoprecipitation of EP300 from both EV and HDAC8 expressing cells and Immunoblotting for total protein acetylation (Fig. 6f). The deacetylation of EP300 was associated with a decrease in its HAT activity (as measured by acetylation of histone H4) in two independent melanoma cell lines (Fig. 6g). Identical experiments following immunoprecipitation of CREBBP did not identify consistent changes in CREBBP acetylation or HAT activity upon expression of HDAC8 (Supplementary Fig. 13c, d). Single locus ChIP assays for NCSC and melanocytic phenotype associated genes showed that increased expression of HDAC8 was associated with increased binding of EP300 to the Jun promoter of *AXL* and a decreased binding of EP300 to the MITF promoter of *MLANA* (Fig. 6h). Together these results demonstrated that HDAC8 modulated EP300 function both through inhibition of its HAT activity and by a switching its association from MITF promoter sites to Jun promoter sites.

### Inhibition of EP300 drives brain metastasis development in melanoma

**Fig. 7:**
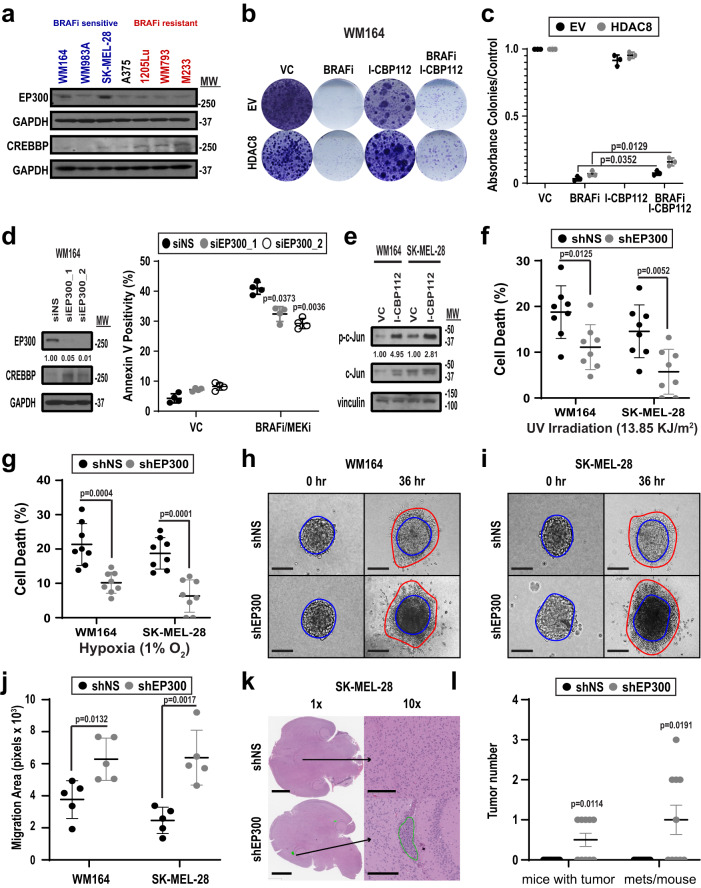
Inhibition of EP300 drives the stress-resistant, invasive phenotype in melanoma cells. **a** Cells were probed for EP300, CREBBP and HDAC8 by immunoblot. **b**, **c** Cells were treated with vemurafenib (1 μmol/L, BRAFi), I-CBP112 (100 nmol/L, EP300i/CREBBPi) or combination for 21 days following acetic acid permeabilization. Significance was determined by a one-way ANOVA followed by a 2-tailed *t* test with *=*p* < 0.05 (EV: *p* = 0.0352, HDAC8: *p* = 0.0129). **d** WM164 cells were transfected with non-silencing siRNA (siNS) or EP300 siRNA (siEP300_1 or siEP300_2). Cells were probed for EP300 and CREBBP after 72 h with ImageJ quantification. Cells were treated with dabrafenib and trametinib (100 nmol/L dabrafenib, 10 nmol/L trametinib, BRAFi-MEKi) for 72 h. Apoptosis was measured by Annexin V APC staining. Significance was determined by a one-way ANOVA followed by a 2-tailed *t* test with *=*p* < 0.05 and **=*p* < 0.01 (siEP300_1: *p* value = 0.0373, siEP300_2: *p* value = 0.0036). **e** Cells were treated with I-CBP112 (100 nmol/L) for 24 h. Lysates were collected and probed for p-c-Jun and c-Jun by immunoblot with ImageJ quantification. Cells were treated with (**f**) 13.85 KJ/m^2^ UV irradiation or (**g**) 1% O2 for 24 h followed by cell death measurement. Significance was determined by a 2-tailed student’s *t* test with *=*p* < 0.05, **=*p* < 0.01 and ***=*p* < 0.001. In (**f**), WM164 *p* value = 0.0125 and SK-MEL-28 *p* value = 0.0052. In g, WM164 *p* value = 0.0004 and SK-MEL-28 *p* value = 0.0001. **h**, **i** Melanoma spheroids infected with shNS or shEP300 were plated in collagen. Scale bars = 500 µm. **j** Invasion area was quantified using ImageJ. Significance was determined by a 2-tailed student’s *t* test with *=*p* < 0.05, and **=*p* < 0.01. (WM164: *p* value = 0.0132 and SK-MEL-28: *p* value = 0.0017). **k**–**l** shNS and shEP300 SK-MEL-28 cells were injected into NOD.CB17-Prkdcscid/J mice and established for 14 days. **k** Brain sections were stained with H&E. Scale bars in 1x images = 4 mm. Scale bars in 10x images = 300 µm. **l** Number of tumors and metastases per mouse were quantified. Significance was determined in 10 mice by a 2-tailed student’s *t* test with *=*p* < 0.05. (tumor #: *p* value = 0.0114 and mouse metastases: *p* value = 0.0191) Experiments were run 3 independent times with an n of 3 in (**c**), 4 in (**d**), 5 in (**j**), and 8 in (**f**) and (**g**). All data are presented as mean values ± SD. Source data are provided as a Source Data file.

Since EP300 is a direct target of HDAC8, we next explored the role of EP300 in driving the stress-resistant, invasive phenotype. We first determined the expression levels of EP300 and CREBBP across a panel of melanoma cell lines that were either sensitive or resistant to BRAFi therapy and found that cell lines that were sensitive to inhibitor therapy had increased EP300 levels (Fig. 7a). Interrogation of the TCGA Melanoma database highlighted a correlation between decreased overall survival and lower levels of EP300 and increased levels of HDAC8, (change of 2-fold expression, *n* = 37) had significantly decreased overall survival compared to patients with unaltered EP300/HDAC8 levels (*n* = 406) (Supplementary Fig. 14a, b). Performing the same analysis with increased levels of HDAC8 and decreased levels of CREBBP did not show a significant decrease in survival (Supplementary Fig. 14c, d). Functional studies were then undertaken to determine if inhibition of EP300 (mimicking its deacetylation by HDAC8) would alter sensitivity of melanoma cells to BRAFi therapy. Co-treatment of *BRAF*-mutant melanoma cell lines with the EP300/CREBBP inhibitor I-CBP112 decreased the sensitivity of the melanoma cells to BRAFi, increasing the number of colonies formed (Fig. 7b, c). Silencing of EP300 through specific siRNA had similar effects and was found to decrease the level of apoptosis induced in response to BRAFi-MEKi therapy (Fig. 7d). The effects were associated with increased c-Jun activity, with EP300 inhibition being found to increase levels of transcriptionally active phospho-c-Jun (Fig. 7e). Silencing of CREBBP by two independent siRNAs led to different levels of knockdown, with one of these be associated with a significant protection from BRAF-MEKi therapy (Supplementary Fig. 15a). Further studies demonstrated that silencing of EP300 through shRNA knockdown (Supplementary Fig. 15b) also protected WM164 and SK-MEL-28 melanoma cells from both UV irradiation and hypoxia (Fig. 7f, g). The converse was also true, with overexpression of EP300 increasing cell death in response to treatment with BRAFi-MEKi (Supplementary Fig. 15c, d), while overexpression of CREBBP did not significantly alter cell death in response to BRAFi-MEKi treatment (Supplementary Fig. 15c, d). As the HDAC8 expressing cells are highly invasive, we next examined if modulating EP300 expression would alter the invasive capacity of melanoma cells. Silencing of EP300 through shRNA knockdown increased the invasion of melanoma cells into 3D collagen matrices (Fig. 7h–j and Supplementary Fig. 16a, b) whereas overexpression of EP300 decreased cell invasion (Supplementary Fig. 17a, b). Accordingly, intracardiac injection of EP300-silenced SK-MEL-28 cells increased the number of mice with brain metastases (5/10 mice with brain mets) compared to the shRNA control (0/9 mice with brain metastases) (Fig. 7k, l and Supplementary Fig. 18a, b). Collectively, our data suggest that HDAC8-mediated deacetylation of EP300 leads to decreased HAT activity, an increase in transcriptionally active JUN and a switch from a melanocytic to a NCSC-like program (model shown in Supplementary Fig. 19).

## Discussion

In the current study we identified a role for HDAC8 in driving a stress-induced transcriptional state in melanoma cells that increases metastasis to the brain. HDAC8 is a type I HDAC that is essential for the correct embryonic development and migration of neural crest cells^32,33^ and has been implicated as a key driver of tumorigenesis in the neural crest tumor neuroblastoma in addition to other tumors such as acute myeloid leukemia and breast cancer^34–36^. HDAC8 is unique among class I HDACs, and differs from HDAC1, 2, 3 in not being phosphorylated and activated by casein kinase 2 (CK2). Instead, HDAC8 is phosphorylated by cAMP-activated PKA signaling at its N-terminus at Serine 39^37^, leading to the inhibition of its HDAC function^38^.

An analysis of a panel of melanoma cell lines identified those with either low or high expression of HDAC8. Cell lines with highest HDAC8 expression had evidence of phenotype switching exemplified by low MITF expression, and this was associated with de novo resistance to BRAFi therapy. By contrast, cell lines with the lowest HDAC8 activity had the highest MITF expression and increased BRAFi therapy sensitivity. HDAC8 expression/activity was also increased in melanoma cells following exposure to multiple stresses including UV irradiation, hypoxia, and drug treatment. Previous studies showed that HDAC8 directly deacetylates p53 under conditions of stress, leading to increased cell survival^39,40^. Increases in both HDAC8 and phospho-c-Jun protein levels were also seen in melanocytes treated with UV irradiation, suggesting a conserved mechanism of stress tolerance between melanocytes and melanoma cells.

An RNA-seq analysis of the HDAC8-driven transcriptional state showed the gene expression profile was akin to the melanoma neural crest stem cell (NCSC)-like state^11,12,16^. ATAC-seq analysis demonstrated increased chromatin accessibility at multiple genes specific to the NCSC state including *FAM83G* and *AXL* in conjunction with decreased accessibility of melanocytic-phenotype genes including *MLANA* and *DCT*. Pathway analysis of the HDAC8-driven state showed an enrichment for pathways involved in focal adhesion, ECM-receptor interactions, the cytoskeleton, proteoglycans, and multiple oncogenic signaling pathways (MAPK, PI3K, RAP1 and Ras). This cell state was associated with increased metastatic potential as HDAC8 expressing cells adopted an amoeboid-like phenotype that was associated with increased invasion into collagen and Matrigel, enhanced transendothelial cell migration and a faster rate of seeding to the lungs. The increased invasion of capacity of the HDAC8-expressing cells was also reflected in an early increase in liver seeding following intracardiac injection and ultimately the enhanced formation of brain metastases.

Metastasis of cancer cells to the brain is a complex, multi-step process^22,24^. Although our studies did not recapitulate the entire brain metastatic cascade, we did model many key steps including (1) survival of the cells in the general circulation, (2) infiltration of the cells into the brain parenchyma and (3) the establishment of macrometastases. The HDAC8-driven transcriptional state likely contributes to several of these processes. First, the effects of HDAC8 on the amoeboid cell state increased the robustness of the melanoma cells (e.g., the cells are more compact with increased mechanical strength) leading to their increased survival under fluid shear stress conditions. Second, the amoeboid phenotype increased invasive capacity allowing the cells to invade endothelial cell monolayers and Matrigel more efficiently, migrate into thick collagen gels and exit the circulation into organs such as the lung. As the brain is a highly protected, difficult to penetrate organ, surrounded by the blood-brain-barrier, it seems likely that melanoma cells with a high invasive capacity (such as those expressing HDAC8) would have a significant advantage in seeding to the brain. A key feature of cancer cells that efficiently form brain metastases is increased expression of Serpins, a family of protease inhibitors that mitigate the deleterious effects of the protease-rich brain microenvironment on tumor cell growth^23^. Analysis of our RNA-seq and ATAC-seq data supported a role for HDAC8 in driving the expression of multiple Serpins. A further adaptation of melanoma cells to the brain is a switch to an oxidative phosphorylation (OXPHOS) metabolic state^10^. In line with this observation, we noted both that introduction of HDAC8 led melanoma cells to adopt an OXPHOS state and that HDAC8 expression was significantly associated with an OXPHOS-like gene signature in a single-cell dataset of human melanoma samples.

Brain metastasis development is often an end stage of melanoma progression^41^. Accordingly, we found that increased expression of HDAC8 in clinical melanoma samples from the TCGA dataset was significantly associated with decreased overall survival. Interrogation of single cell RNA-seq data from a cohort of melanoma brain and leptomeningeal metastasis specimens revealed a correlation between expression of HDAC8 and the NCSC transcriptional signature. These findings agree with a recent multi-omic study of melanoma brain metastases that demonstrated the cancer cells adopted a neuronal-like state when growing in the brain^42^. Although some brain metastasis samples did show high expression levels of HDAC8, not all did, suggesting that HDAC8 may not be an absolute requirement for the maintenance of established brain metastases. In addition, analysis of skin metastasis samples identified the presence of cells that expressed HDAC8. It thus seems that HDAC8 drives a cell state that permits the increased seeding to and initial survival of melanoma cells in the brain, potentially through the combined effects of increased invasive capacity, enhanced cell survival, metabolic adaptations and the expression of microenvironmental modulators (such as the Serpins).

HDACs exert their effects through deacetylation of both histone and non-histone targets. Our ChIP-seq and ATAC-Seq analyses demonstrated HDAC8 to have little effect upon global chromatin accessibility or promoter/enhancer activity. Specific changes were however noted at discrete transcriptional sites, with in-depth analysis showing increased accessibility at AP-1, Jun, ATF3, FRA1 and TEAD sites. Less chromatin accessibility was seen at promoter sites for melanocytic-cell state associated transcription factors, such as MITF. ATAC-Seq analysis showed that accessibility to TEAD1 and TEAD4 binding sites increased upon expression of HDAC8 with ChIP-Seq suggesting increased promoter activity of TEAD-target genes. Many of the genes associated with the neural crest-like phenotype appear to be controlled by both TEAD and c-Jun transcription factors, indicating both transcription factor families may be under the regulation of HDAC8. These findings are supported by previous work showing that both AP-1/Jun and TEAD’s have also are important drivers of BRAFi resistance in melanoma^43,44^.

As ChIP-Seq analysis and immunoblotting for total histone acetylation did not suggest any global changes in histone acetylation following HDAC8 introduction we reasoned that non-histone targets of HDAC8 could be responsible for driving the transcriptional program we observed. Mass spectrometry-based proteomics used to map changes in protein acetylation showed that HDAC8 altered the acetylation of multiple proteins implicated in the regulation of lipid metabolism. The link between HDAC8 and lipid metabolism is intriguing given recent evidence demonstrating that HDAC8 is a key regulator of fatty acid acylation^45^. Of relevance to the gene expression program identified, we identified transcriptional co-regulators whose acetylation was modulated by HDAC8. One of the central hubs in the transcriptional regulator network was EP300 (E1A-binding protein, KAT3B) a histone acetyl transferase (HAT) with a high degree of homology to CREB-binding protein (CREBBP, CBP, KAT3A)^46,47^. Both EP300 and CREBBP are HATs that primarily catalyze the acetylation of histone H3 at lysine 27 (H3K27ac)^47^, which acts to mark enhancer regions in the genome that form critical loci for the recruitment of master transcription factors. Increased expression of HDAC8 decreased H3K27 acetylation marks at the promoters of multiple melanocytic state genes including *MLANA* and *DCT*, and reduced chromatin access and promoter activity at melanocyte lineage transcription factor sites including MITF. While EP300 and CREBBP have similar homology and overlapping functions, they are also known to have distinct roles, depending upon the cellular context. In hematopoietic cells, CREBBP was required for proliferation and self-renewal, while EP300 was critical for differentiation and expression of genes important for cell cycle progression and DNA repair^48,49^. H3K27ac ChIP-Seq studies have also shown distinct genome-wide binding patterns for EP300 and CREBBP, indicating EP300 and CREBBP regulate the enhancers of different genes^50,51^. Our data suggest that HDAC8 selectively inhibits EP300 and not CREBBP, helping to explain why specific genetic regions are affected without changes in global H3K27 acetylation.

In-depth proteomic analysis identified 4 lysine residues K1542, K1546, K1549, and K1550 in the HAT domain of EP300 that were deacetylated following introduction of HDAC8. EP300 HAT domain residues, including K1542 and K1549, have previously been shown to be deacetylated by the NAD^+^-dependent deacetylase sirtuin 2 (SIRT2), resulting in alterations in EP300 autoacetylation and protein binding^52^. Decreased acetylation of EP300 inhibited its HAT function in in vitro acetylation assays. Our data agree with previous studies showing that hypoacetylation of the EP300 activation loop, consisting of amino acids 1523-1554, decreases EP300 HAT activity^53^.

Recent work has additionally suggested that EP300 directly acetylates MITF, leading to altered transcription factor distribution^31^. EP300 also acetylates c-Jun, leading to decreased DNA binding, increased turnover and decreased transcription of JUN targets^54,55^. It is likely that the decrease in EP300 HAT activity following HDAC8 upregulation we observed would decrease MITF acetylation leading to a reduction of its transcriptional activity (as our ATAC-seq and RNA-seq data suggested). By contrast, inhibition of EP300 had opposite effects on Jun, with its deacetylation being associated with an increase in transcriptionally activated (phosphorylated) Jun and increased H3K27ac and chromatin accessibility at Jun promoters/genes. To further investigate the effects of EP300 inactivation in driving the invasive phenotype we undertook phenotypic studies and found that both genetic and pharmacological inhibition of EP300 increased melanoma cell survival and mediated resistance of melanoma cells to BRAFi therapy, hypoxia and UV irradiation. In addition to this, inhibition of EP300 also mimicked HDAC8 activation by promoting melanoma cell invasion and by increasing the formation of brain metastases in mice.

We have demonstrated that stress-mediated activation of HDAC8 leads to a switch from MITF- to Jun-driven transcriptional programs in melanoma cells that leads to the adoption of a transcriptional state with characteristics of the previously reported NCSC state. The deacetylation of Jun increases its phosphorylation and transcriptional activity, leading to the initiation of an Jun/AP-1 driven, NCSC-like transcriptional program. At the same time, HDAC8-mediated deacetylation of EP300 inhibits its HAT function, decreasing accessibility at MITF promoter sites and altering the distribution of H3K27 at key melanocytic genes. Induction of this program by HDAC8 leads to the melanoma cells adopting a highly resilient, amoeboid phenotype that drives invasion and survival under both shear stress conditions and in the brain parenchyma. This HDAC8-driven program was also observed in melanocytes exposed to UV irradiation, providing a link between a conserved melanocyte survival program and the aggressive metastatic behavior of melanoma cells.

## Methods

Our research complies with all relevant ethical regulations. All animal studies were approved by the University of South Florida’s Institutional Animal Care and Use Committee (#IS00010687).

### Cell culture and reagents

The FOM173, NHEM, 1205Lu, A375, M233, SK-MEL-28, WM164, WM793, and WM983A cell lines were a generous gift from Dr. Meenhard Herlyn (The Wistar Institute, Philadelphia, PA). HERMES1 and HERMES3 cell lines were acquired from Dr. Dorothy Bennett (St. Georges Hospital Medical School, UK). Human umbilical vein endothelial cells (HUVEC, # PCS-100-013) were acquired from ATCC (Manassas, Virginia). HERMES1 and HERMES3 were maintained in 10% RPMI containing 200 nM 12-O-tetradecanoyl phorbol 13-acetate (Sigma), 200 pM cholera toxin (Sigma), 10 ng ml−1 human stem cell factor (R&D) and 10 nM endothelin 1 (Sigma). All other melanoma and melanocyte cell lines were maintained in RPMI media (Corning Inc., Corning, NY) containing 5% BSA (Millipore Sigma, Burlington, MA) and a 1:10,000 dilution of plasmocin (InvivoGen, San Diego, CA). HUVEC cells were maintained in Endothelial Cell Growth Medium (Cell Applications Inc, San Diego, CA). Every 3 months, cells were tested for *Mycoplasma* contamination using the Plasmotest-Mycoplasma Detection Test (InvivoGen) with the last test date on 06/20/2023. Cell lines were authenticated by ATCC’s Human STR human cell line authentication service and cell lines were replaced from frozen stocks after 10 passages. HUVEC cells were discarded after 3 passages. The reagents dabrafenib, vemurafenib, trametinib, and I-CBP112 were ordered from Millipore Sigma.

### Generation of plasmids and transfection

HDAC8 and its corresponding EV control plasmid were a generous gift from Dr. Edward Seto and were described previously^38^. EP300 siRNA (siEP300_1: SASI_Hs01_00052818 and siRNA_2: EHU155151), EP300shRNA (TRCN0000009883), and non-silencing (NS) shRNA (SHC016V) was from Millipore Sigma and non-targeting control siRNA was ordered from Thermo Fisher. For transfection, cells were placed in OPTI-MEM media in the presence of the plasmid and Lipofectamine 2000 (Thermo Fisher Scientific, Waltham, MA). Transfection was achieved following the manufacturer’s protocol. Transfected HDAC8 and EV cells were maintained in hygromycin (Thermo Fisher Scientific). shNS and shEP300 expressing cells were maintained in puromycin (Millipore Sigma).

### Immunoblotting and immunoprecipitation

Lysates were acquired and immunoblots were performed as previously described^20^. The anti-HDAC8 antibody was described in^38^. Antibodies against SMC3 (# 5696, D47B5), p-EGFR (Y1068, # 3777, D7A5), EGFR (#4267, D38B1), p-c-Jun (S73, #3270, D47G9), c-Jun (# 9165, 60A8), HDAC1 (#34589, D5C6U), HDAC2 (#57156, D6S5P), HDAC3 (#85057, D2O1K), acetyl-histone 3 (Lys27, #8173, D5E4), histone3 (#4499, D1H2), EP300 (# 86377, D8Z4E), and CREBBP (# 7389, D6C5) were purchased from Cell Signaling Technologies (Danvers, MA). An anti-acetyl-SMC3 antibody was a kind gift from Forma Therapeutics. An Anti-MITF (NB110-10872) antibody was ordered from Novus (Littleton, CO). Anti-vinculin (V9131), anti-GAPDH (G9545) antibodies were from Millipore Sigma. For immunoblots, all primary antibodies were used at a 1:1000 dilution.

Lysates for immunoprecipitation assays were collected and assayed as previously described^20^. Briefly, 500 µg of protein was incubated with an anti-EP300 antibody (CST, # 86377, D8Z4E,1:100 dilution) or an IgG control (CST, #3900, DA1E, 1:100) under constant rotation overnight at 4 degrees. Lysates were incubated with Protein A/G agarose (Thermo Fisher Scientific) for 4 h followed by isolation of beads by centrifugation. The resulting protein/bead complexes were disassociated with loading buffer before being collected and probed with an anti-acetyl antibody (CST, #9441) by immunoblot. Immunoblot and immunoprecipitation protein levels were quantified using ImageJ Software. All immunoblots were normalized to loading controls for analysis and immunoprecipitations were normalized to input controls.

### Cell viability assays

Cells were treated with drug for 72 h, collected and stained with Annexin V APC (BD biosciences, Franklin Lakes, NJ). Fluorescence was read on a FACSCanto (BD biosciences) and apoptotic cells were measured for Annexin V positive populations using FloJo software (v10.7.1).

To measure cell death following UV irradiation and hypoxia, 300 cells were analyzed for cell death using a 0.4% trypan blue solution (Thermo Fisher Scientific) 24 h after stress induction. Hypoxic conditions were maintained in a Hypoxia Incubator Chamber (STEMCELL Technologies, Vancouver, Canada) for 24 h at 94% N_2_, 1% O_2_ and 5% CO_2_. Cells were UV-irradiated for 15 min (13.85 KJ/m^2^) using a UV Solar Simulator UV SOL (Newport Technologies, Irvine, CA) with the UV generated from the simulator consisting of 8.0% UVB and 92.0% UVA. Cells were incubated for 24 h and cell death was measured.

For long term viability following continuous vemurafenib exposure, a colony formation assay was performed. Briefly, 1000 cells were placed in a 6 well plate. Cells were treated with drug for 21 days, replacing drug and media twice weekly, before being stained with a 0.5% Crystal Violet solution (1:1 water to methanol) for 4 h under constant agitation. Colonies were quantified for absorption at 580 nm following acetic acid permeabilization.

For shear stress viability, 10,000 melanoma cells were plated on a 0.4 mm channel µ-Slide I Luer (Ibidi, Martinsried, Planegg, Germany). The cells were incubated at 37 °C for 24 h with a constant 10 dyne/cm2 unidirectional flow using the Ibidi perfusion pump system. Cells were subsequently stained with Calcein AM (Molecular Probes, Eugene, OR) and PI. Cells were imaged using an EVOS 5100 autofluorescence imager (Thermo Fisher Scientific).

### Adhesion/Migration assays

For 3D spheroid assays, 10,000 cells were incubated on agar for 72 h to form spheroids. Cells were then transferred to a Type 1 Bovine Collagen matrix (Advanced Biomatrix, Carlsbad, CA) as previously described^56^. Cells migrated for 36–48 h and images were taken using a Nikon Eclipse Ci-L fluorescent microscope (Tokyo, Japan) or EVOS 5100 autofluorescence imager.

Confocal invasion assays were performed as previously described previously in^56^. Briefly, Cells were plated onto Matrigel (BD biosciences) coated transwell inserts and allowed to invade for 48 h. Cells were fixed and stained with phalloidin-AF594 (Molecular Probes) and imaged with a Zeiss confocal microscope (20x) at 0 μm with 0.5 μm image slices taken throughout the distance of invasion.

For transendothelial migration assays, HUVEC cells were plated and grown to confluence in transwell inserts as previously described^56^. 10,000 DiI-((2*Z*)−2-[(*E*)−3-(3,3-dimethyl-1-octadecylindol-1-ium-2-yl)prop-2-enylidene]−3,3-dimethyl-1-octadecylindole; perchlorate) stained cells were allowed to migrate through the HUVEC coated membrane for 1 h. Non-migratory cells were removed followed by imaging with an EVOS 5100 autofluorescence imager. For collagen adhesion assays, 20,000 cells were grown on a Type I Bovine Collagen/PBS (pH 7.0) matrix for 48 h. Cells were subsequently imaged using a Nikon Eclipse Ci-L fluorescent microscope.

### HAT activity assay

To measure EP300 activity, a Histone Acetyltransferase Activity Assay Kit (cat. ab239713, Abcam, Cambridge, United Kingdom) was used following the manufacturers protocol. For collection of the lysates, 500 µg of protein was immunoprecipitated with an anti-EP300 or IgG control. Fluorescence was read (Excitation 535 nm, Emission: 587 nm) at 35 and 40 min and data were analyzed following manufacturers protocol.

### RNA sequencing and data analysis

Total RNA was extracted with the Direct-zol^TM^ RNA MiniPrep Plus Kit (Zymo Research, Irvine, CA). Library preparation and sequencing were performed by Novogene using TruSeq RNA Library Preparation Kit and NovaSeq 6000 (Illumina, San Diego, CA) with PE150. Short reads were filtered and trimmed using Trimmomatic (v 0.36). QC on the original and trimmed reads was performed using FastQC (v 0.11.4) and MultiQC (v 1.1). The reads were aligned to the transcriptome using STAR (v 2.7.3a). Transcript abundance was quantified using RSEM (v 1.2.31). Differential expression analysis was performed using DESeq2, with an FDR-corrected *P*-value threshold of 0.05 and a log_2_fold change of ±0.58. Gene ontology analysis of biological processes was performed using Enrichr and Gene set enrichment analysis (GSEA).

### ChIPmentation sequencing and data analysis

Chromatin immunoprecipitation coupled with next-generation sequencing (ChIP-Seq) was performed with a reference exogenous genome in combination with Tagmentation for library preparation as previously described^57^. Cell lines were cross-linked with 0.8% formaldehyde and quenched with 0.125 M glycine. Cells were lysed (10% glycerol, 1% Triton TX-100, 0.1 mM EDTA, 14 mM NaCl 25 mM Hepes pH 7.5), washed and 10 × 10^6^ cell nuclei were resuspended in 1 ml sonication buffer (1 mM EDTA, 10 mM Tris-HCl pH 7.5). Nuclei were sonicated using a Covaris E220 Focused-ultrasonicator (Covaris Inc., Woburn, MA) for 30 min (15% Duty Cycle, 200 cycles/burst, and Peak incidence 140). After pre-clearing with Protein A/G Dynabeads (Invitrogen), DNA were immunoprecipitated using antibodies including anti-H3K27ac (Active Motif, #39133, Carlsbad, CA), anti-H3K27me3 (CST, #9733), and IgG (CST, #3900) overnight at 4 °C. Precipitated chromatin was washed and incubated in Tagmentation buffer containing Tn5 transposase enzyme (Illumina). Samples were reverse crosslinked for 4 h at 65 °C, followed by DNA purification and amplification for the library with index primers using KAPA HiFi HotStart Kit (Roche, Indianapolis, IN). The libraries were purified, size-selected for between 200 bp and 700 bp using Ampure beads XP (Beckman Coulter, Brea, CA), and pooled for Novaseq 6000 S4 2×150 flow cell (ICBR Next-Gen Sequencing Core, University of Florida). The input sequences were trimmed using Trimmomatic. Quality control was performed before and after trimming using FastQC. The input sequences were then aligned to the GRCh38 genome using Bowtie (v 2.3.5.1). Peak detection was performed using MACS (v 2.1.2) and motif finding on peak regions was performed with the HOMER find-Motif’s function.

### ATAC sequencing and data analysis

Nuclei were isolated from 200,000 cells and ATAC-Seq was performed on aliquots of 50,000 nuclei as previously described^58^. ATAC-seq libraries were purified by size selection using Ampure XP beads (Beckman Coulter, Brea, CA), and library quality assessed by fragment analyzer and qPCR for GAPDH and a genomic desert region. Sequencing of pooled libraries was performed on a Novaseq 6000 S4 2×150 flow cell (ICBR Next-Gen Sequencing Core, University of Florida). Reads were trimmed using Trimmomatic (v 0.36), and QC on the original and trimmed reads was performed using FastQC (v 0.11.4) and MultiQC (v 1.1). The reads were aligned to the human genome version GRCh38 using Bowtie (v. 2.3.3), and ATAC peak calling was performed using the MACS (v 2.1.2). Differential peak analysis was performed using DASA (https://github.com/uf-icbr-bioinformatics/dasa)^59^. Custom scripts were used to produce heatmaps and enrichment plots.

### ChIP assay

ChIP was adapted from the manufacturers protocol (Rockland, Pottstown, PA). 20 million cells were cross-linked with 1.0% formaldehyde (30 min) and quenched with 0.125 M glycine. Cells were Dounce homogenized and extracted chromatin were sonicated using a pulse sonicator (Thermo Fisher Scientific, 15 s at 50% on, 1 min off, 12 times). Samples were pre-cleared with Protein A/G Agarose and a 1/20 aliquot was saved as input control. DNA were immunoprecipitated using an anti-EP300 (ab14984, Abcam) and anti-IgG2a (ab18413, Abcam) antibody overnight at 4 °C. Following a 4-h protein A/G incubation, samples were de-crosslinked at 65 degrees C for 5 h.

DNA was purified and subsequently amplified using the following primers:

Axl: 5ʹ GCTCACATTCTCAGCATTCC 3ʹ

3ʹ GGAAGACCTGGTGAGCATGC 5ʹ

MLANA: 5’ GCTGTAACAGAGCTGTAACAGAGC 3ʹ

3ʹ GGAGTTGTTGAATCAGCTGCC 5ʹ

DNA was run on a 7900HT Fast Real-Time PCR System (Thermo Fisher Scientific) for 40 cycles using TaqMan master mix (Applied Biosystems, Waltham, MA). Samples were normalized to 5% input control.

### scRNA-Seq data analysis

To investigate the relationship between the NCSC signature, the OXPHOS signature and HDAC8 expression in tumor cells, we performed principal component analysis (PCA) of the neural crest stem cell-like signature^11^ or OXPHOS signature^10^ (total 131 genes from KEGG_OXIDATIVE_PHOSPHORYLATION) using single cell RNA-seq (scRNA-Seq) data generated from 9,862 tumor cells by 10X Genomics platform from 43 melanoma patient specimens obtained from brain, skin or leptomeningeal sites^28^. The first principal component score (PC1 score) was used as surrogate of two gene signatures. The t-SNE projection was used to visualize the HDAC8 gene expression and PC1 score of the neural crest stem cell-like signature at single cell level. HDAC8 mean expression (in log2 normalized scale) was used to summarize the HDAC level for each patient sample. A Spearman correlation analysis was performed to investigate the correlation between the mean of PC1 score for each signature and mean HDAC8 expression in melanoma cell compartment from (1) the 43 patient samples or (2) tumor cells from each of the three different metastatic sites, respectively.

### Acetylomics

Cells were lysed in denaturing lysis buffer containing 8 M urea, 20 mM HEPES (pH 8), 1 mM sodium orthovanadate, 2.5 mM sodium pyrophosphate and 1 mM β-glycerophosphate. The proteins were reduced with 4.5 mM DTT and alkylated with 10 mM iodoacetamide. Trypsin digestion was carried out at room temperature overnight, and tryptic peptides were then acidified with aqueous 1% trifluoroacetic acid (TFA) and desalted with C18 Sep-Pak cartridges according to the manufacturer’s procedure (WAT051910, Waters Corp, Milford, MA). The peptides were then frozen on dry ice before lyophilization. Following lyophilization, the dried peptide pellet was re-dissolved in immunoaffinity purification (IAP) buffer containing aqueous 50 mM MOPS pH 7.2, 10 mM sodium phosphate and 50 mM sodium chloride. Acetyl lysine-containing peptides were immunoprecipitated with immobilized anti-Acetyl-Lysine Motif (Ac-K) antibody (#13416, CST). The acetyllysine peptides were eluted twice with aqueous 0.15% trifluoroacetic acid (TFA). The eluted acetylated peptides were labeled with TMT 10plex reagents: triplicate HDAC8 overexpression were labeled in 127 N, 127 C and 128 N channels. Triplicate empty vector controls (EV) were labeled in 129 C, 130 N and 130 C channels. Mixed peptides were fractionated with the Pierce High pH Reversed Phase Kit (ThermoFisher 84868), and nine fractions were collected according to the manufacturer’s protocol. A nanoflow ultra-high performance liquid chromatograph (RSLC, Dionex, Sunnyvale, CA) interfaced with an electrospray bench top quadrupole-orbitrap mass spectrometer (Orbitrap Exploris480 with FAIMS, Thermo) was used for liquid chromatography tandem mass spectrometry (LC-MS/MS) peptide sequencing experiments. The sample was first loaded onto a pre-column (C18 PepMap100, 100 µm ID x 2 cm length packed with C18 reversed-phase resin, 5 µm particle size, 100 Å pore size) and washed for 8 min with aqueous 2% acetonitrile containing 0.04% trifluoroacetic acid. The trapped peptides were eluted onto the analytical column, (C18 PepMap100, 75 µm ID x 25 cm length, 2 µm particle size, 100 Å pore size, Thermo). The 120-min gradient was programmed as: 95% solvent A (aqueous 2% acetonitrile + 0.1% formic acid) for 8 min, solvent B (90% acetonitrile + 0.1% formic acid) from 5 to 38.5% in 90 min, then solvent B from 50% to 90% B in 7 min and held at 90% for 5 min, followed by solvent B from 90% to 5% in 1 min and re-equilibration for 10 min. The flow rate on analytical column was 300 nl/min. Spray voltage was 2100 V. Capillary temperature was set at 300 °C. For field asymmetric waveform ion mobility spectrometry (FAIMS), two compensation voltage (CV) values were applied: −45 V and −65 V. Data-dependent scans were performed following each survey scan using 15 s exclusion for previously sampled peptide peaks using 1.5 s cycle time before the next survey scan. MS and MS/MS resolutions were set at 120,000 and 45,000, respectively. Automatic gain control was set to 100%, and ion accumulation time was set to automatic. Database searches were performed with MaxQuant^60^. Enzyme specificity was set as trypsin with up to 4 missed cleavages. Variable modifications included lysine acetylation, tandem mass tag labeling of N-termini and lysines, carbamidomethylation of cysteine, and methionine oxidation. Mass measurement error was set to 4.5 ppm for peptides and 20 ppm for fragment ions. False discovery rate was set to 0.5% at the peptide level. Values were log(2) transformed and normalized using iterative rank order normalization (IRON)^61^. Significant changes in acetylated proteins peptides were determined to be greater than ±2 standard deviations away from the average of the data set with *t* test *p* values below 0.05. Gene ontology of significantly deacetylated proteins were carried out using the STRING protein-protein interaction network.

### In vivo assays

All animal studies were approved by the University of South Florida’s Institutional Animal Care and Use Committee (#IS00010687). In all in vivo experiments, a tumor burden resulting in less than 20% animal weight loss for 72 h, tumor volume below 1500 mm^3, and tumor size below 2 cm was permitted. The maximum tumor burden was not exceeded. 1 million WM164_HDAC8, WM164_EV, SK-MEL-28_EV, SK-MEL-28_HDAC8, SK-MEL-28_shNS, SK-MEL-28_shEP300, 1205Lu_EV, and 1205Lu_HDAC8 expressing cells were introduced into 10 week old NOD.CB17-Prkdcscid/J female mice (Jackson laboratories, Strain #:001303, Bar Harbor ME) by intracardiac injection. For time course experiments, tumors were allowed to invade for 5, 10, 15, and 20 days with mice euthanized at each timepoint. For brain metastasis studies, tumors were allowed to migrate to the brain for 14 days and mice were subsequently euthanized. After euthanasia, brains, livers and lungs were collected and fixed in formalin for 24 h followed by storage in 70% ethanol. The collected organs were paraffin embedded. Sections were H&E stained and subjected to immunohistochemistry (IHC) with an anti-gp100 (ab787, Abcam, 1:100) antibody. Slides were subsequently imaged and tumors quantified using Aperio Imagescope.

### Statistical analysis and reproducibility

For cell death assays, stress assays, ChIP assay and in vivo metastatic analyses, a 1-way ANOVA was performed followed by a two-tailed post-hoc students *t* test. with non-significance equaling *p* > 0.05 and *=*p* < 0.05, **=*p* < 0.01, and ***=*p* < 0.001. For migration assays and the HAT activity assay, a two-tailed student’s *t* test was used. Significance was determined with non-significance equaling *p* > 0.05 and significance equaling *=*p* < 0.05, **=*p* < 0.01, and ***=*p* < 0.001. Significance in in vivo assays was determined by a chi-squared test for differences in tumor between groups. For reproducibility, all in vitro assays excluding sequencing assays (ChIP-Seq, ATAC-Seq, RNA-Seq, scRNA-Seq, acetylomics) were run 3 independent times. All in vivo assays were run 2 independent times.

### Reporting summary

Further information on research design is available in the Nature Portfolio Reporting Summary linked to this article.

### Supplementary information

Supplementary Information

Description of Additional Supplementary Files

Supplementary Data 1

Supplementary Data 2

Reporting Summary

### Source data

Source Data

## Data Availability

The raw sequence reads, raw counts and differential expression for RNA-seq data generated in this study have been deposited in the Gene Expression Omnibus (GEO) [(GSE218625) and (GSE240307]). The raw sequence reads for ATAC-Seq and ChIP-Seq data generated in this study have been deposited with links to BioProject accession number PRJNA903203 in the NCBI BioProject database [https://www.ncbi.nlm.nih.gov/bioproject/?term=prjna903203]. The raw reads for the proteomic data generated in this study have been deposited in the ProteomeXchange Consortium via the PRIDE partner repository with the dataset identifier PXD044471. The raw data files for the previously published and publicly available scRNA-seq data analyzed in this study have been deposited in the Gene Expression Omnibus (GSE174401). Source data are provided with this paper.

## Code availability

The Differential peak analysis was performed using DASA (https://github.com/uf-icbr-bioinformatics/dasa). The code for DASA is available at 10.5281/zenodo.8381172^59^.
