# Supplementary material for: HDAC8-mediated inhibition of EP300 drives a transcriptional state that increases melanoma brain metastasis
Source: Nat Commun. 2023 Nov 29;14:7759. doi: 10.1038/s41467-023-43519-1 (PMC10686983; doi:10.1038/s41467-023-43519-1)
Supplement: Supplementary file 1 — Supplementary Information [file 41467_2023_43519_MOESM1_ESM.pdf]

Supplementary Information:

Plasmids

A pcDNA3.1-p300 plasmid was a gift from Warner Greene (Addgene plasmid # 23252). A CREBBP plasmid (NM_004380) and the corresponding EV Control plasmid was ordered from Origene (Rockland, MD). siRNAs against CREBBP (SR423497) were ordered from Origene. An incucyte GFP containing plasmid was ordered from Sartorius (Gottingen, Niedersachsen, Germany).

Immunoblotting

Immunoblots probing for histone markers were imaged using a Li-Cor Odyssey CLx Imaging System (Li-Cor). For histone markers, nuclear extracts were acquired using an Active Motif Nuclear protein extraction kit. An anti-HDAC8 (Ab187139) and anti-histone 4(Ab7311) antibody was ordered from Abcam. Beta-tubulin (86298s), Histone-H3 (4499s) and a histone-H4 (2935c) antibody were ordered from Cell Signaling Technology. H3K9ac antibody (61251) was ordered from Active Motif. A H4K16ac antibody (MA5-27794) was ordered from Invitrogen while a H4K20ac antibody (13-0039) was ordered from EpiCypher.

Proliferation assays

For mTT assays, 10,000 EV and HDAC8 expressing cells were treated with indicated concentrations of vemurafenib (BRAFi) for 72 hours. After 72 hours, cells were incubated with 0.5 mg/ml mTT dye and subsequently solubilized with Sorensen's buffer/DMSO. Plates were read at an absorbance of 560 nm.

For cell proliferation assays 100,000 EV and HDAC8 expressing cells were plated and allowed to proliferate for 24, 48, 72, and 96 hours. Live cells were counted using a Countess automatic cell counter at indicated time points.

Patient database analysis

Patient samples containing RNA expression data in the TCGA PanCancer Atlas melanoma dataset were interrogated for gene expression of HDAC8, EP300 and CREBBP. For the HDAC8/EP300 and HDAC8/CREBBP survival analyses, a z-score of +1 was used for HDAC8 in addition to a z-score of -1 used for EP300 or CREBBP compared to all samples.

Oxygen Consumption assays

SKMEL-28 (1.5x104 cells/well) and WM164 (1.0x104 cells/well) were plated overnight in the Seahorse 96-well cell culture plate. Oxygen consumption rate (OCR) was measured using the XF Cell Mito Stress Test Kit on the Seahorse XFe96 instrument (Agilent, Santa Clara, CA) following manufacturer’s protocol. Data represents the mean of two independent experiments, obtained from 6 technical replicates within each experiment. Student’s t-test was performed on each of the 3 readings after FCCP injection.

In vivo assays

For tail vein injection assays, 1 million GFP expressing WM164_HDAC8 and WM164_EV cells were introduced into NOD.CB17-Prkdcscid/J mice by tail vein injection. Tumors were allowed to invade for 10 hours or 24 hours. Lungs were subsequently extracted and fixed for 24 hours. Whole lungs were imaged by confocal microscopy for presence of GFP. For IHCs, an anti-CD31 antibody (ab281583) was used from Abcam.

a

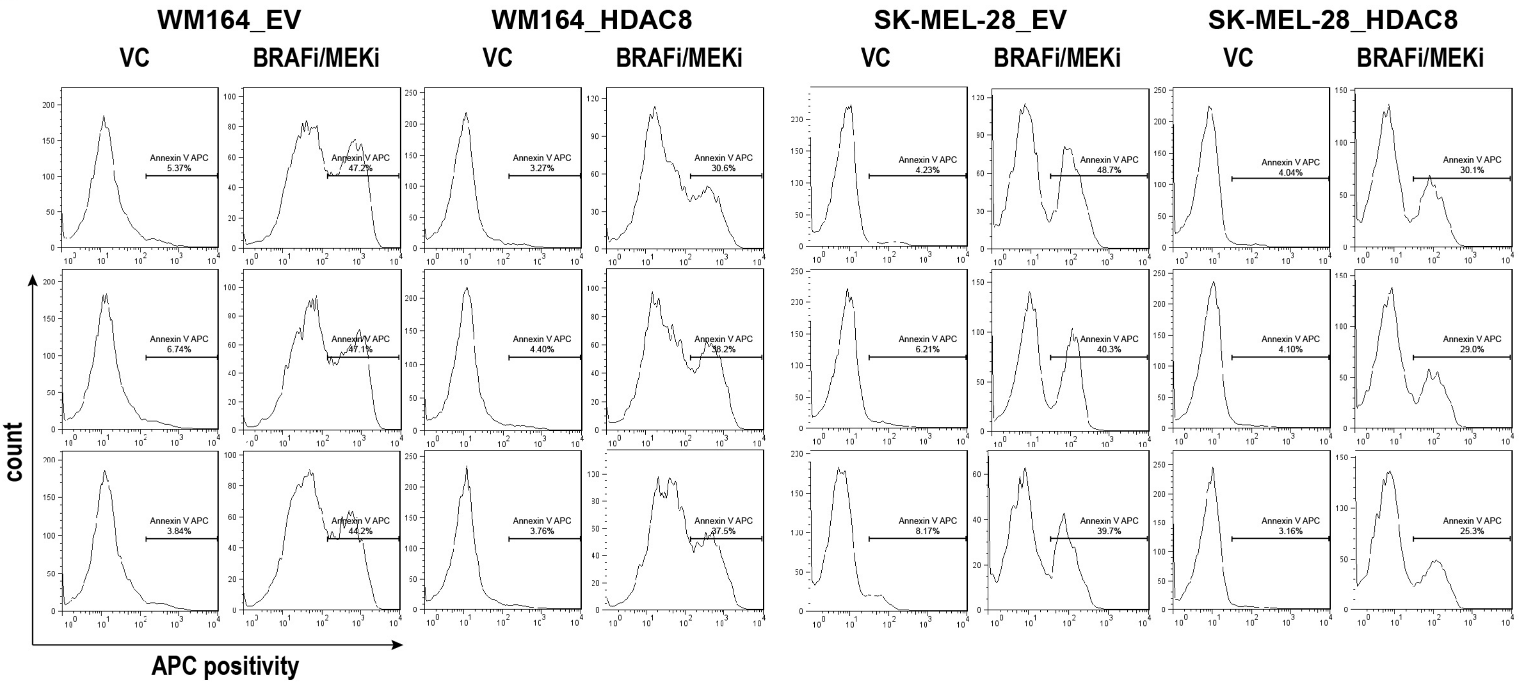

b

UV irradiation

n	WM164_EV live cells	WM164_EV dead cells	WM164_EV % dead	WM164_HDAC8 live cells	WM164_HDAC8 dead cells	WM164_HDAC8 % dead	SK-MEL-28_EV live cells	SK-MEL-28_EV dead cells	SK-MEL-28_EV % dead	SK-MEL-28_HDAC8 live cells	SK-MEL-28_HDAC8 dead cells	SK-MEL-28_HDAC8 % dead
1	76	21	27.6	69	2	2.9	62	15	24.2	58	6	10.3
2	65	20	30.7	58	3	5.2	71	18	25.4	61	6	9.8
3	80	19	23.8	65	0	0	60	16	26.7	69	8	11.6
4	75	30	40	70	7	10	84	24	28.6	60	4	6.7
5	58	21	36.2	75	7	5.2	74	14	18.9	54	5	9.3
6	79	19	24.1	64	7	10.9	62	12	19.4	68	10	14.7
7	74	14	18.9	61	8	13.1	86	15	17.4	74	8	10.8
8	102	22	21.6	58	9	15.5	67	10	14.9	82	5	6.1

hypoxia

n	WM164_EV live cells	WM164_EV dead cells	WM164_EV % dead	WM164_HDAC8 live cells	WM164_HDAC8 dead cells	WM164_HDAC8 % dead	SK-MEL-28_EV live cells	SK-MEL-28_EV dead cells	SK-MEL-28_EV % dead	SK-MEL-28_HDAC8 live cells	SK-MEL-28_HDAC8 dead cells	SK-MEL-28_HDAC8 % dead
1	83	16	19.3	76	4	5.3	78	7	9.0	84	9.5	10.3
2	103	13	12.6	59	0	0	93	13	14.0	94	8.5	9.8
3	96	7	7.3	67	9	13.4	75	18	24.0	74	2.7	11.6
4	65	21	32.3	68	0	0	83	15	18.1	62	4.8	6.7
5	72	15	20.8	80	8	10.0	92	21	22.8	65	0	9.3
6	70	8	11.4	62	0	0	76	16	21.1	80	6.3	14.7
7	97	28	28.9	74	0	0	64	19	29.7	78	14.1	10.8
8	83	15	18.1	93	9	9.7	69	22	31.9	62	0	6.1

c

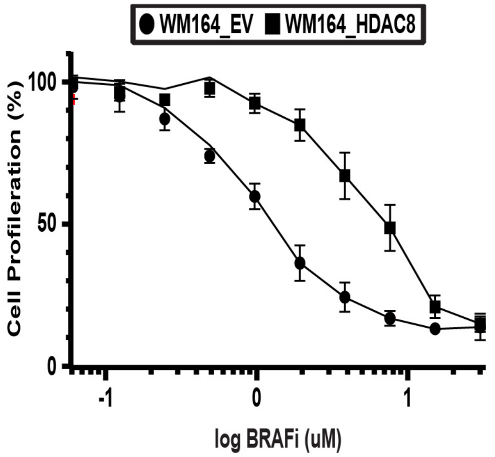

d

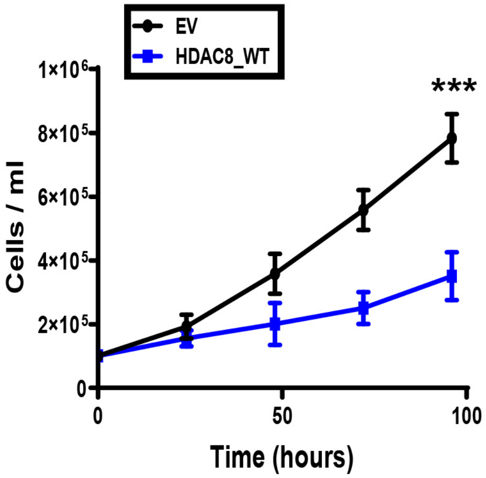

e

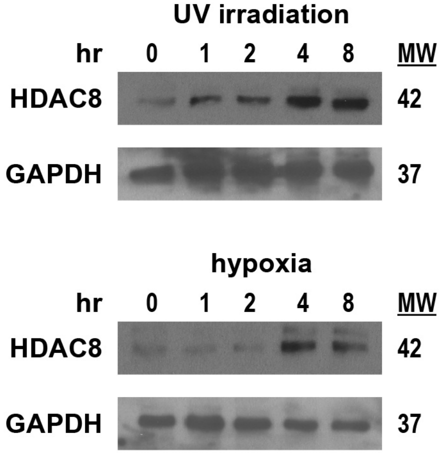

Supplemental Figure 1: HDAC8 expression increases resistance to stress.
a: Raw data images for Annexin V APC experiments done in figure 1c are shown. **b:** Cell counts for trypan blue experiments done in figure 1d and 1e are shown. **c:** 10,000 EV and HDAC8 expressing cells were treated with indicated concentrations of vemurafenib (BRAFi) for 72 hours. After 72 hours, cells were incubated with 0.5 mg/ml mTT dye and subsequently solubilized with Sorensen's buffer/DMSO. Plates were read at an absorbance of 560 nm. **d:** 100,000 EV and HDAC8 expressing cells were plated and allowed to proliferate for indicated time points. Live cells were counted using a Countess automatic cell counter. Significance was determined by a one-way ANOVA followed by a post hoc t-test with ***=p<0.005. **e:** WM164 cells were treated with 13.85 KJ/m^2 UV irradiation or 1% O_2 for indicated times. Cells were probed for HDAC8 and GAPDH protein expression. Source data are provided as a Source Data file.

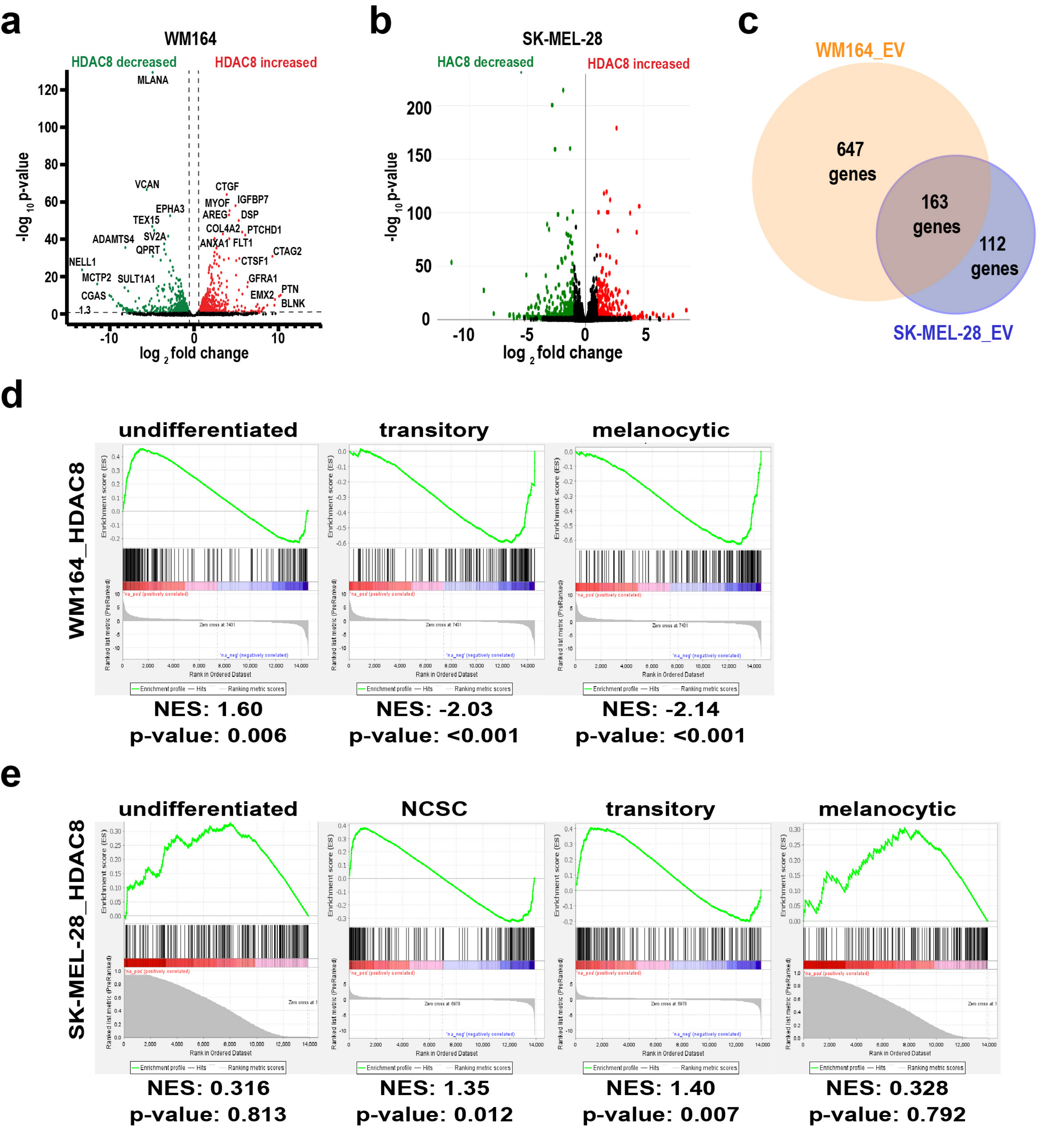

Supplemental Figure 2: HDAC8 expression enhances expression of NCSC genes.
a-b: RNA-seq was performed on EV and HDAC8 expressing (a) WM164 and (b) SK-MEL-28 cell lines with significantly changed genes assigned a log2 fold change value +/- 0.58 and a -log10 p-value above 1.3. c: Cell lines were compared to find common genes with enhanced expression in EV cells compared to HDAC8 expressing cells. d: A Ranked Order analysis was performed on indicated cell lines using undifferentiated, transitory, and melanocytic genesets processed by GSEA software. e: A Ranked Order analysis was performed on indicated cell lines using undifferentiated, NCSC, transitory, and melanocytic genesets processed by GSEA software. Shown is the normalized enrichment score (NES) and 2-sided nominal p-value of the dataset without multiple hypothesis testing. Significance is determined by having a nominal p-value<0.05.

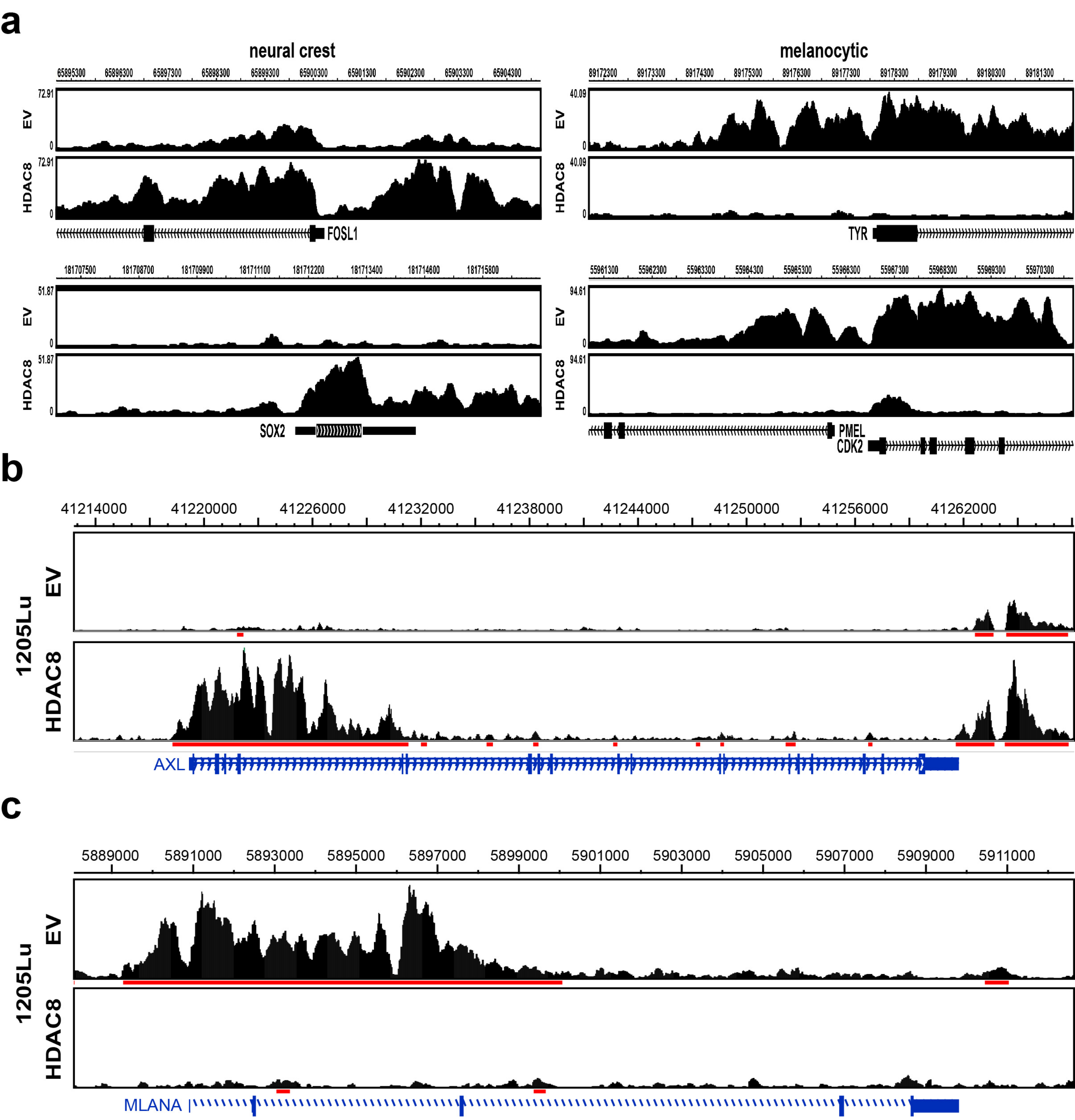

Supplemental Figure 3: HDAC8 expression enhances H3K27 binding in NCSC gene promoter regions. a-c: ChIP-Seq was performed on EV and HDAC8 expressing WM164 and 1205Lu cells using an acetyl-H3K27 antibody followed by interrogation of gene promoter regions. a: Shown are promoter regions for representative neural crest and melanocytic genes in WM164 cell lines. b: Shown is the promoter region for the neural crest/undifferentiated gene AXL in 1205Lu_EV and 1205Lu_HDAC8 expressing cells. c: Shown is the promoter region for the melanocytic gene MLANA in 1205Lu_EV and 1205Lu_HDAC8 expressing cells.

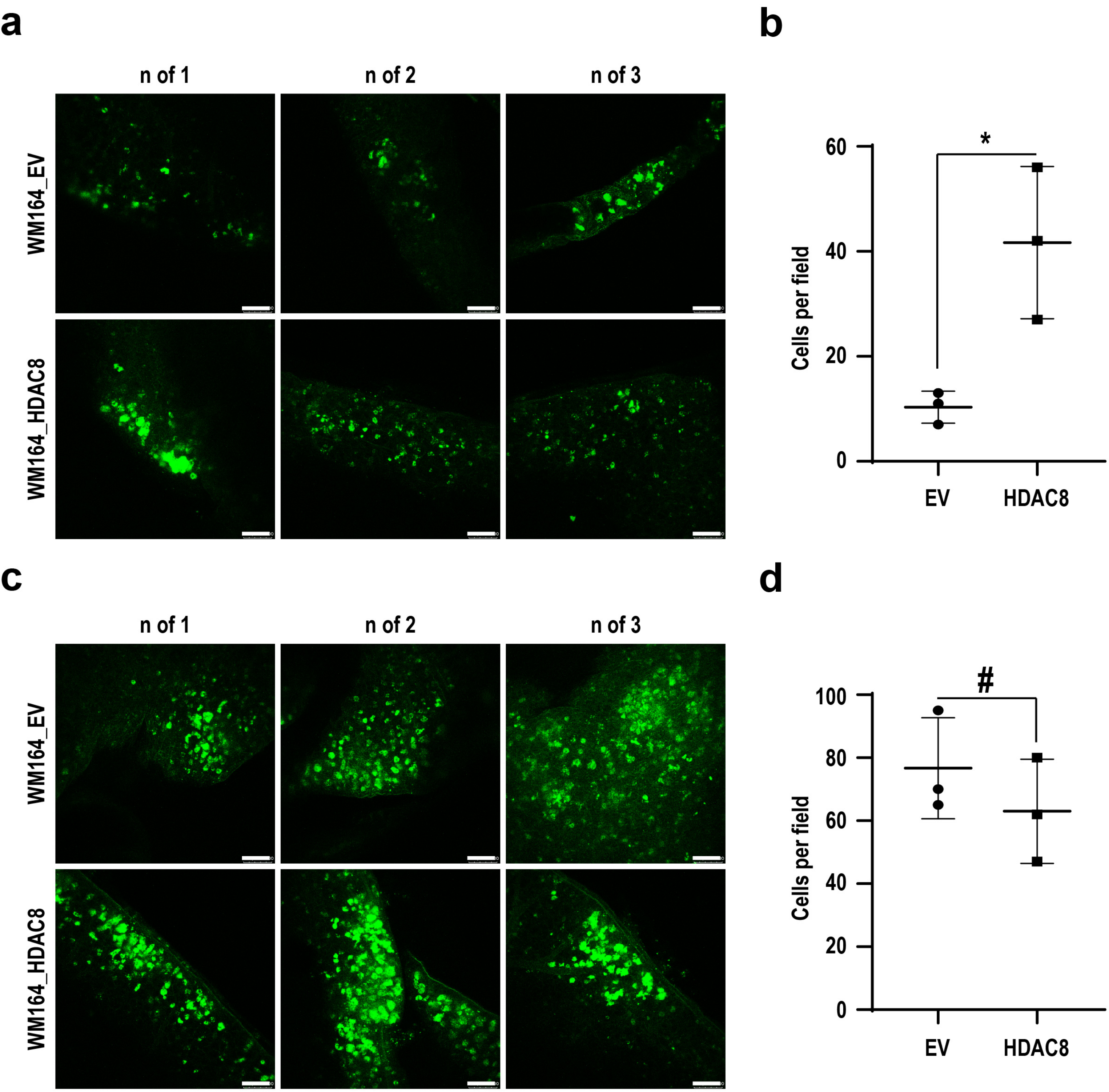

Supplemental Figure 4: HDAC8 expression increases invasion *in vivo.*
a: GFP expressing WM164_HDAC8 and WM164_EV cells were introduced into NOD.CB17-Prkdcscid/J mice by tail vein injection. Tumors were allowed to invade for 10 hours. Lungs were extracted and imaged by confocal microscopy. Scale bars=100 µm. b: Images were quantified for number of images per field using ImageJ. c: GFP expressing WM164_HDAC8 and WM164_EV cells were introduced into NOD.CB17-Prkdcscid/J mice by tail vein injection. Tumors were allowed to invade for 24 hours. Lungs were extracted and imaged by confocal microscopy. Scale bars=100 µm. d: Images were quantified for number of images per field using ImageJ. Significance was determined by a 2-sided student's t-test with *=p<0.05 and #=p>0.05 (8 hr: p-value=0.0288). All experiments were run 2 independent times with an n of 3 in each cohort. All data are presented as mean values +/- SD. Source data are provided as a Source Data file.

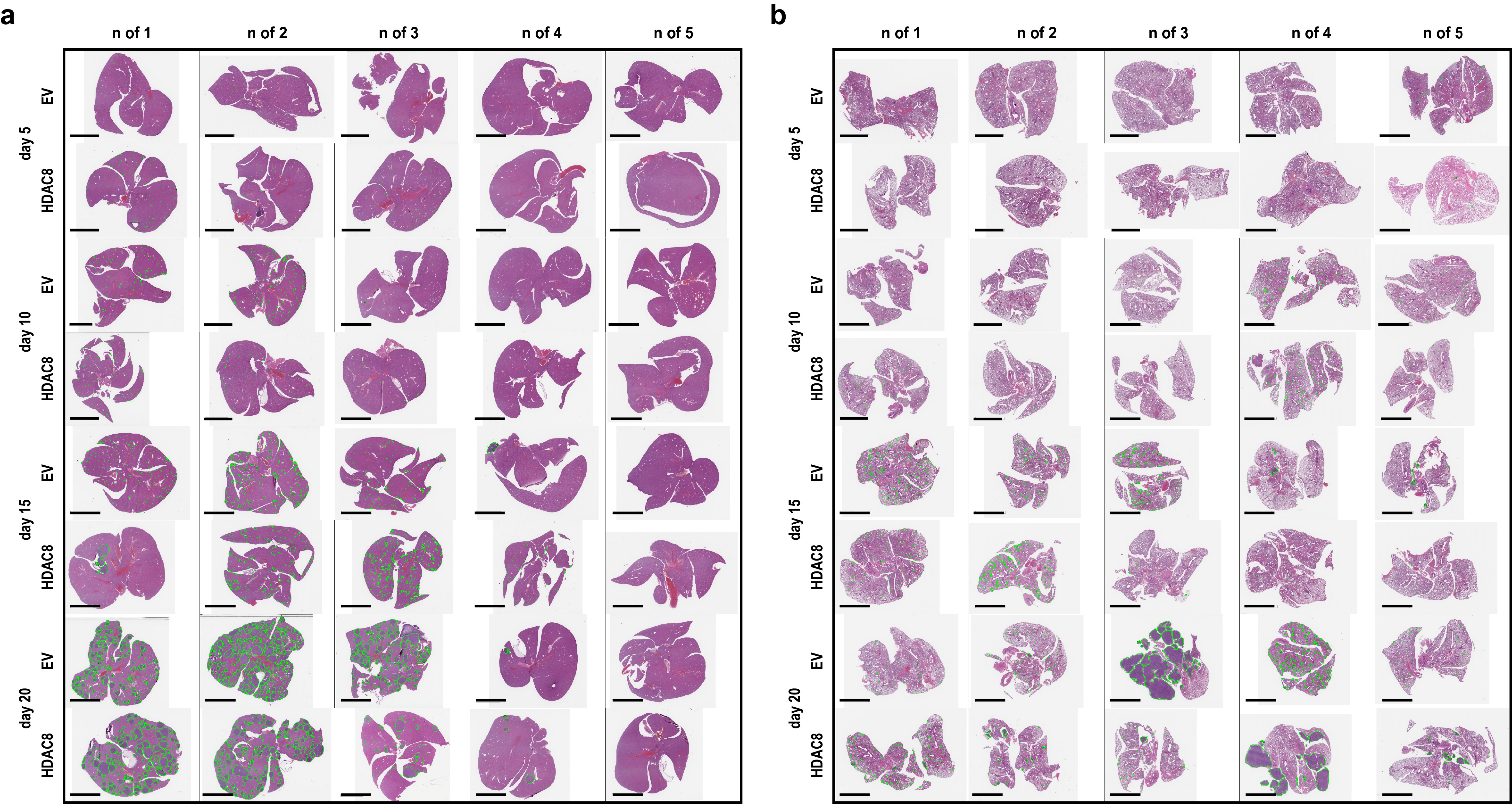

Supplemental Figure 5: HDAC8 expression does not increase metastasis to the liver or lungs
WM164_HDAC8 and WM164_EV expressing cells were introduced into NOD.CB17-Prkdcscid/J mice by intracardiac injection. Tumors were allowed to establish for indicated time-points. a: Livers were extracted and stained for tumors by H&E. Scale bars=6 mm. b: Lungs were extracted and stained for tumors by H&E. Scale bars=5 mm.

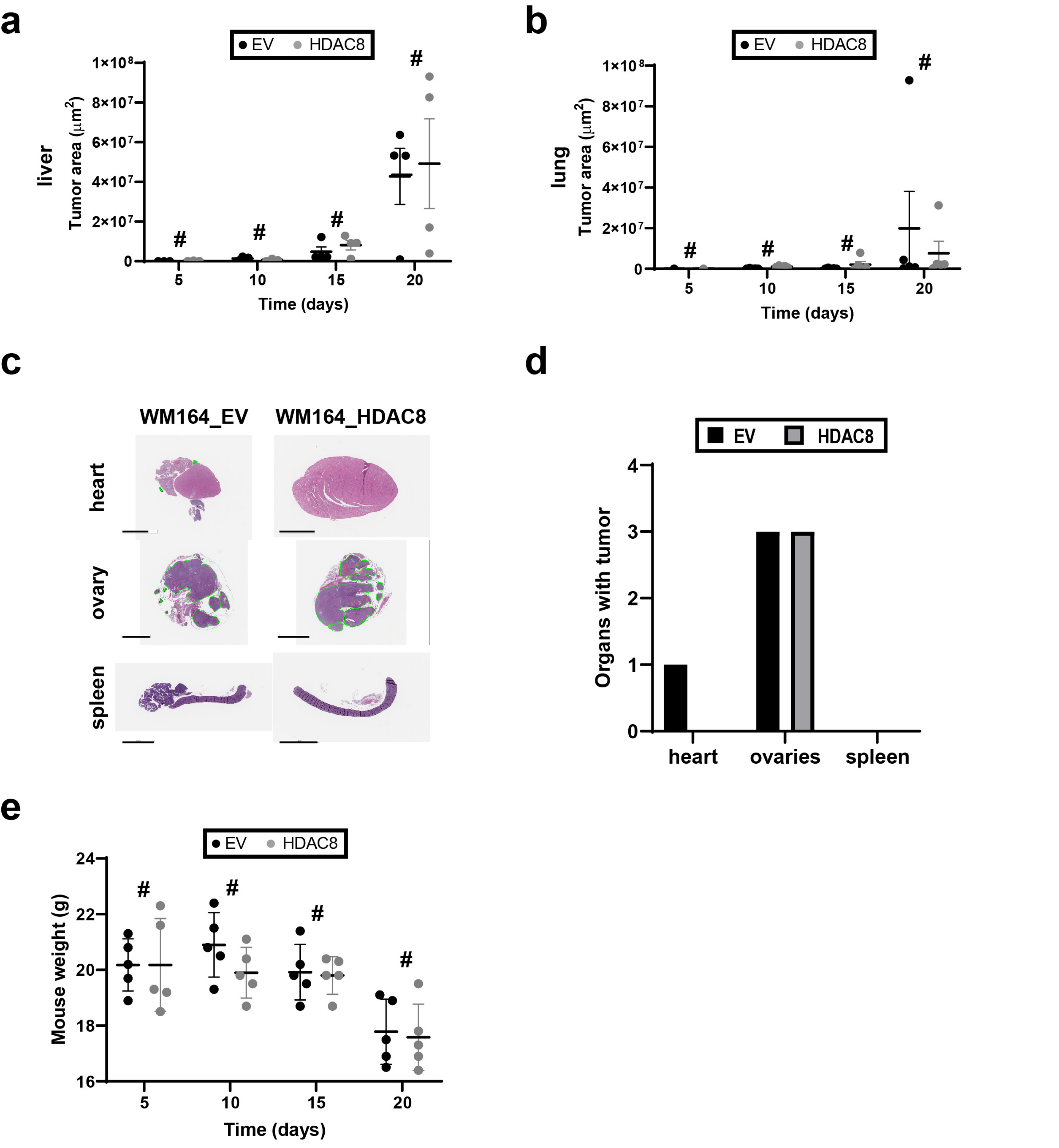

Supplemental Figure 6: HDAC8 expression does not enhance tumor growth in the liver or lungs.
a: Liver H&E sections represented in 4a were quantified for tumor volume per mouse using Imagescope. b: Lung H&E sections represented in 4b were quantified for tumor volume per mouse using Imagescope. c-d: WM164_HDAC8 and WM164_EV expressing cells were introduced into NOD.CB17-Prkdcscid/J mice by intracardiac injection. Tumors were allowed to establish for 20 days. Hearts, ovaries, and spleens were collected and stained by H&E. **c:** Representative H&E images are shown. Heart scale bars=2 mm. Ovary scale bars=4 mm. Spleen scale bars=3 mm d: Number of collected organs containing tumors is shown. e: Mouse weights were recorded at endpoint. Significance in (a), (b), and (e) was determined by a one-way ANOVA followed by a post hoc t-test with #=p>0.05. All experiments were run 2 independent times with an n of 5 in each cohort. All data are presented as mean values +/- SD. Source data are provided as a Source Data file.

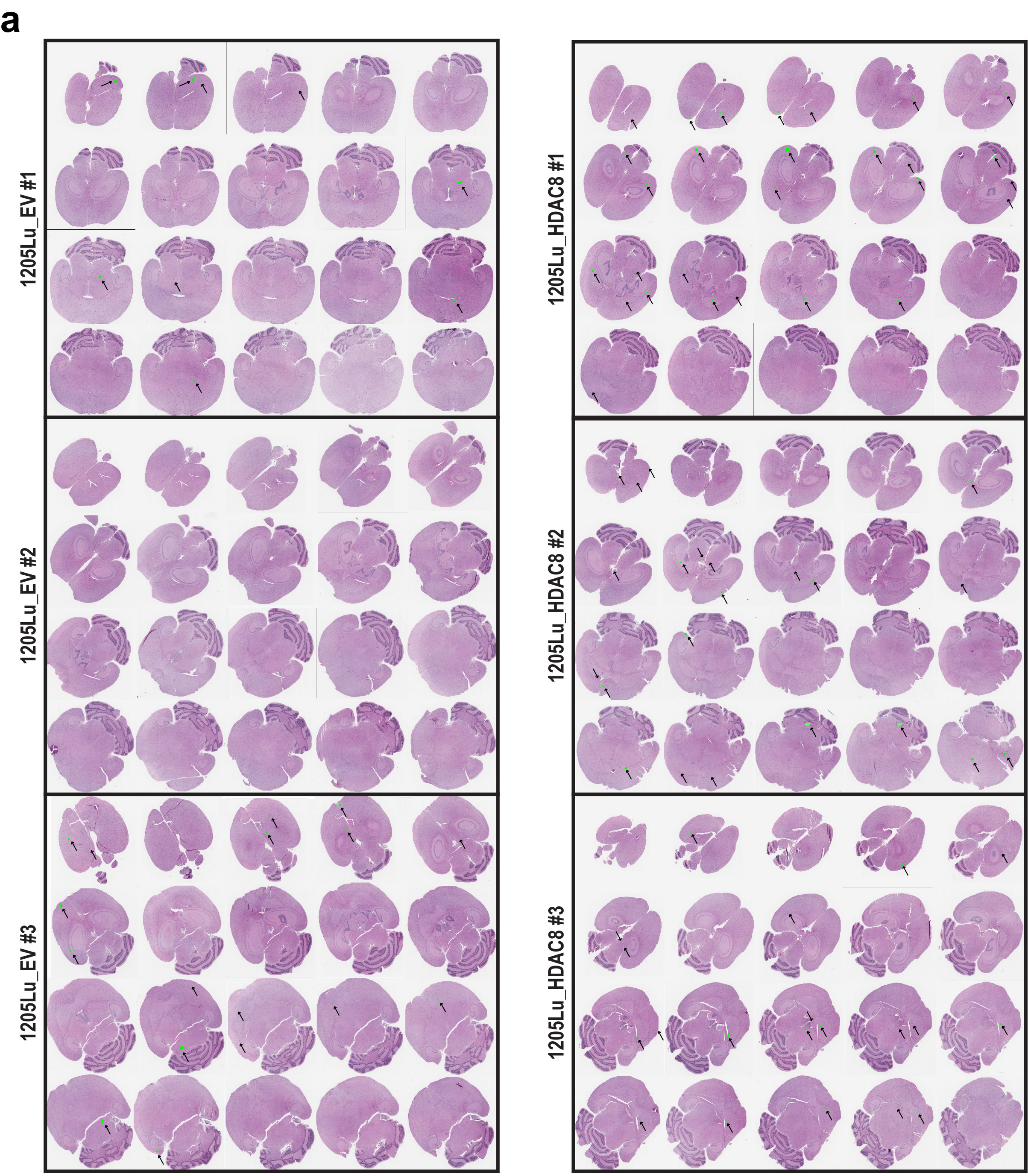

Supplemental Figure 7: HDAC8 increases brain metastasis formation.
a: 1205Lu_HDAC8 and 1205Lu_EV expressing cells were introduced into NOD.CB17-Prkdcscid/J mice by intracardiac injection. Tumors were allowed to establish for 14 days. 3 brains for indicated cells were sectioned 150 microns apart and stained by H&E. Shown are 20 images for each brain. Tumors are indicated in green and highlighted with arrows.

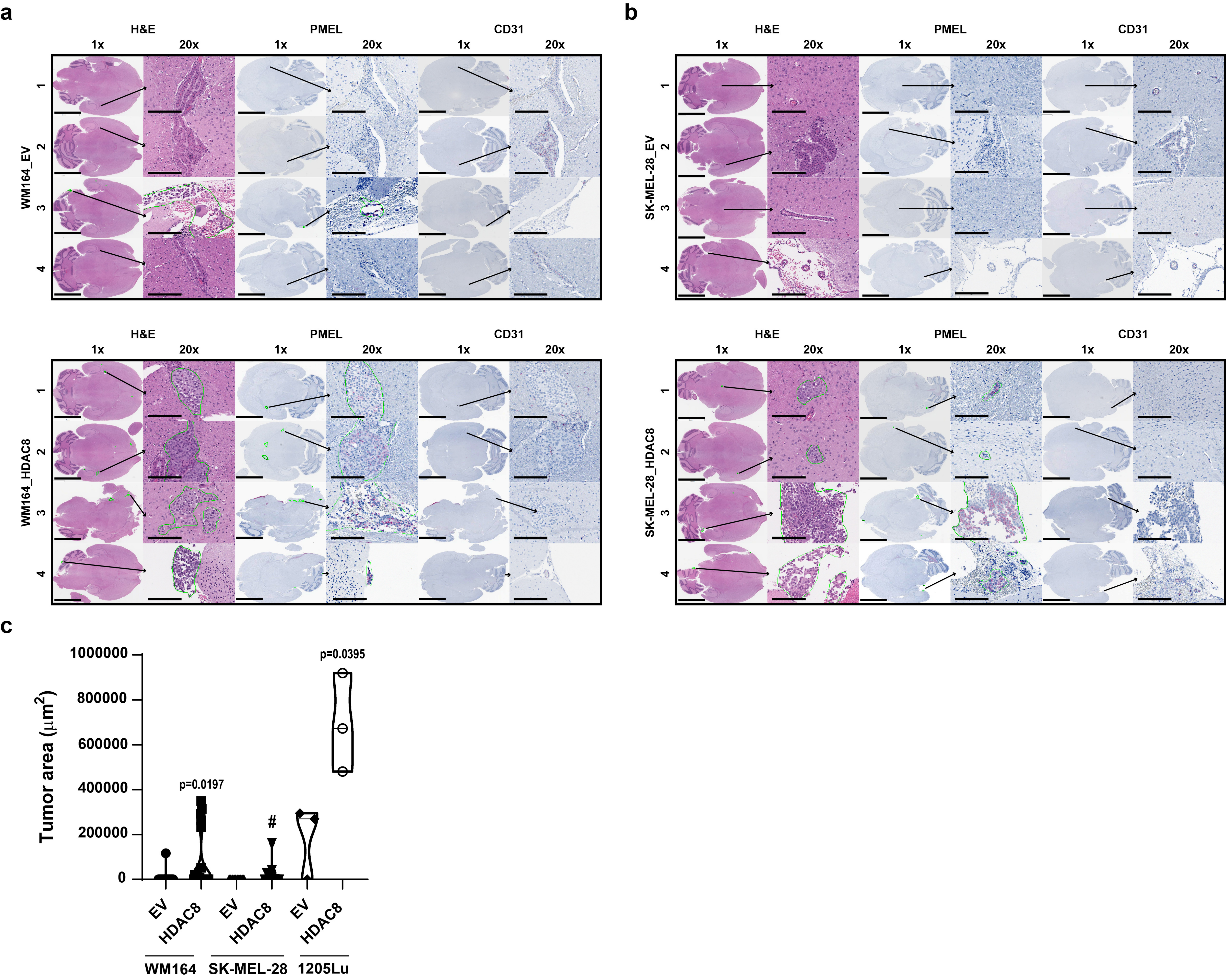

Supplemental Figure 8: HDAC8 expression increases metastasis to the brain

a: WM164_HDAC8 and WM164_EV expressing cells were introduced into NOD.CB17-Prkdcscid/J mice by intracardiac injection and allowed to invade for 14 days. Brains were stained with H&E along with IHCs using PMEL and CD31. 1x scale bars=4 mm. 10x scale bars=200 μm. b: SK-MEL-28_HDAC8 and SK-MEL-28_EV expressing cells were introduced into NOD.CB17-Prkdcscid/J by iintracardiac injection and allowed to invade for 14 days. Brains were stained with H&E along with IHCs using PMEL and CD31. 1x scale bars=4 mm. 10x scale bars=200 μm. c: Tumor areas were quantified in Imagescope using *in vivo* brain sections collected from indicated cell lines. Significance was determined by a 2-tailed student's t-test with *=p<0.05 and #=p>0.05 (WM164: p-value=0.0197, SK-MEL-28: p-value=0.1057, 1205Lu: p-value=0.0395). All experiments were run 2 independent times with an n of 20 in WM164 experiments, an n of 10 in SK-MEL-28 experiments and an n of 3 in 1205Lu experiments. Source data are provided as a Source Data file.

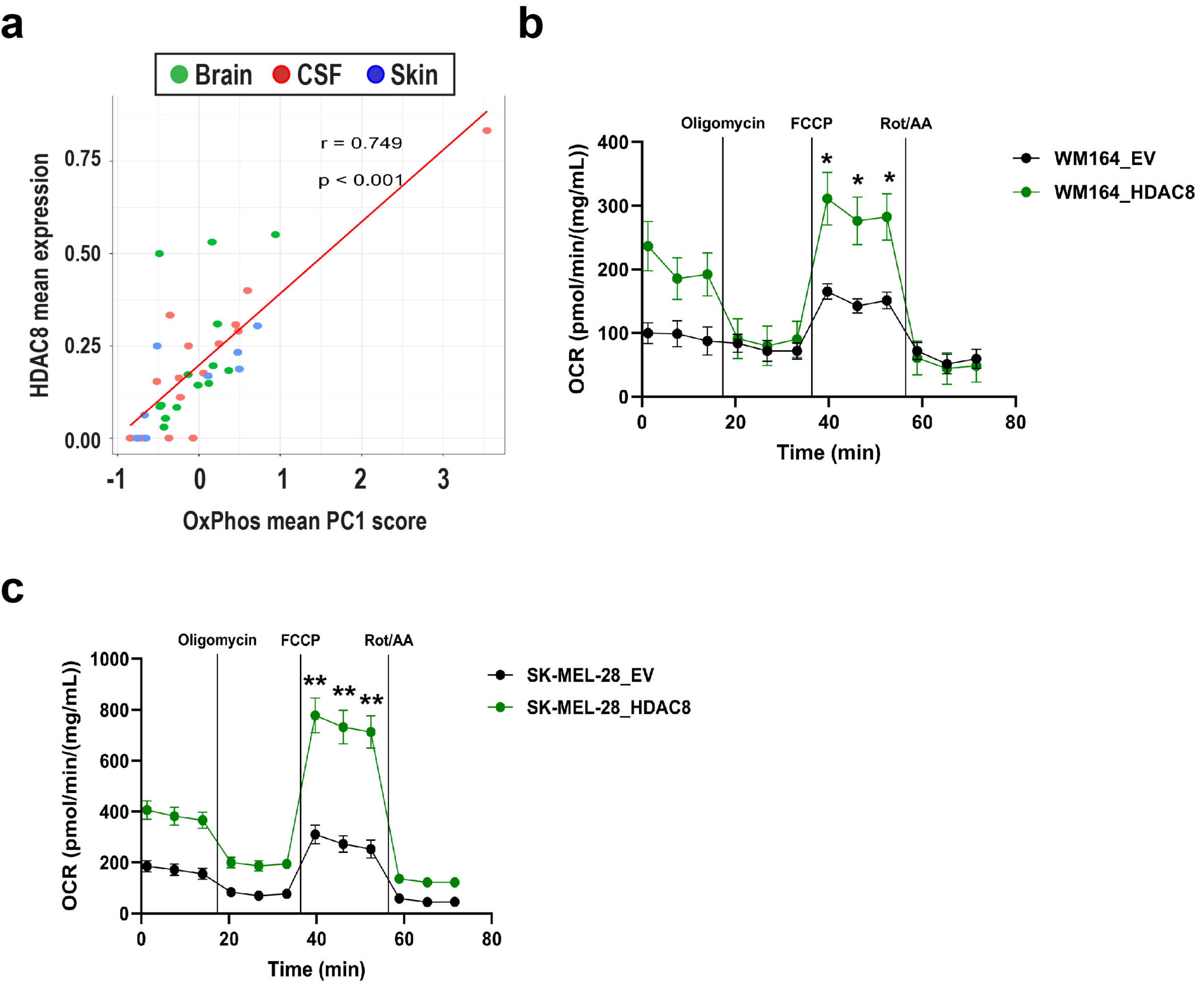

Supplemental Figure 9: HDAC8 expression leads to higher oxygen consumption

a: Patient-derived scRNA-Seq data (Skin=melanoma cutaneous samples, CSF=melanoma CSF samples, MB: melanoma brain metastases samples) was interrogated for expression of oxidative phosphorylation (OxPhos) genes as determined by the KEGG geneset. A Spearman correlation analysis was run to determine the relationship of HDAC8 expression and a OxPhos phenotype in the scRNA-Seq database. b-c: (b) WM164 (1.0x104 cells/well) and (c) SK-MEL-28 (1.5x104 cells/well) cells were plated overnight in the Seahorse 96-well cell culture plate. Oxygen consumption rate was measured over time. Significance in (b) and (c) was determined by a one-way ANOVA followed by a post hoc t-test with *=p<0.05 and **=p<0.01. Experiments were run 2 independent times with an n of 3 in each cohort. All data are presented as mean values +/- SEM. Source data are provided as a Source Data file.

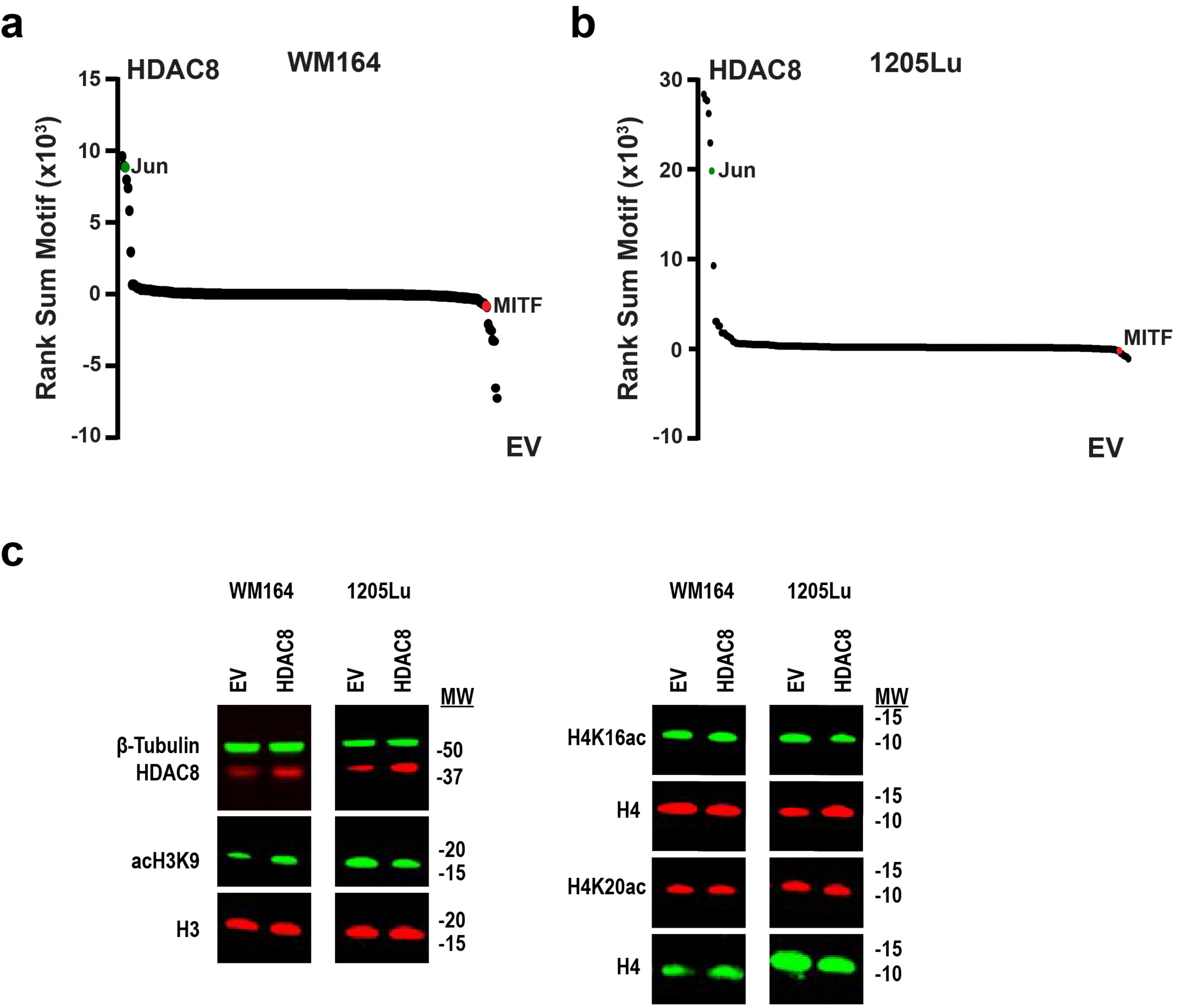

Supplemental Figure 10: HDAC8 expression increased accessibility at Jun binding sites and decreased accessibility at MITF binding sites.
a-b: ATAC-Seq was carried out on HDAC8 and EV expressing (a) WM164 and (b) 1205Lu cell lines. A HOMER analysis was performed on chromatin accessibility transcription factor binding motifs. Differences in accessibility between HDAC8 and EV cells for available motifs were graphed based on rank sum differences in p-value. c: Indicated cell lines were probed for HDAC8, acH3K9, total H3 histone, acH4K16, acH4K20, and total H4 histone protein expression by immunoblot. Immunoblots were run for 3 independent experiments. Source data are provided as a Source Data file.

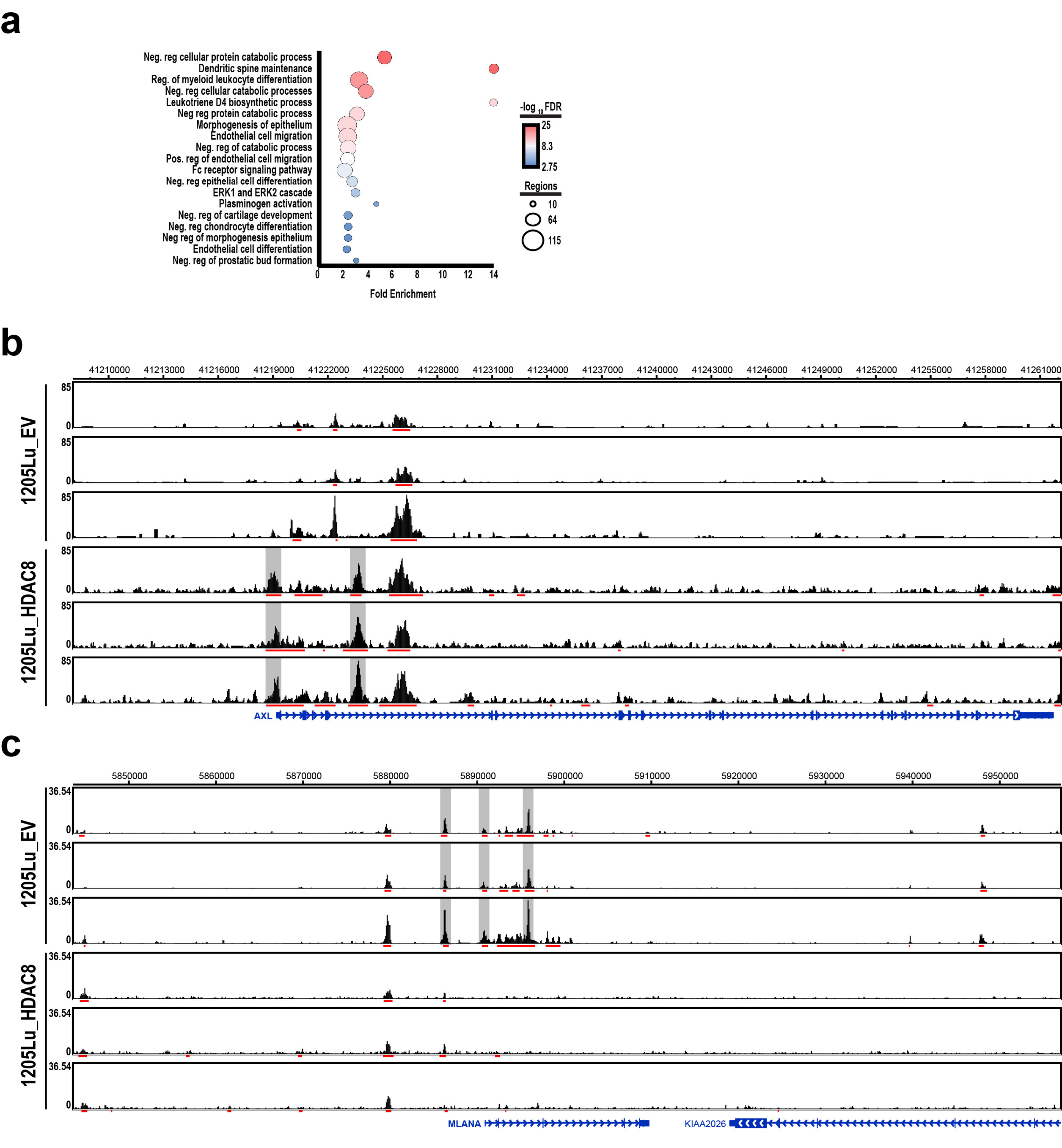

Supplemental Figure 11: HDAC8 expression changes chromatin accessibility at neural crest and melanocytic regions in melanoma.

a: A GREAT analysis was performed on genes with enhanced accessibility/H3K27 acetylation around their transcription start sites in EV cells compared to HDAC8 expressing cells. b-c: ATAC-Seq tracks from 1205Lu_EV and 1205Lu_HDAC8 cells showing chromatin accessibility around (b) neural crest and (c) melanocytic genes.

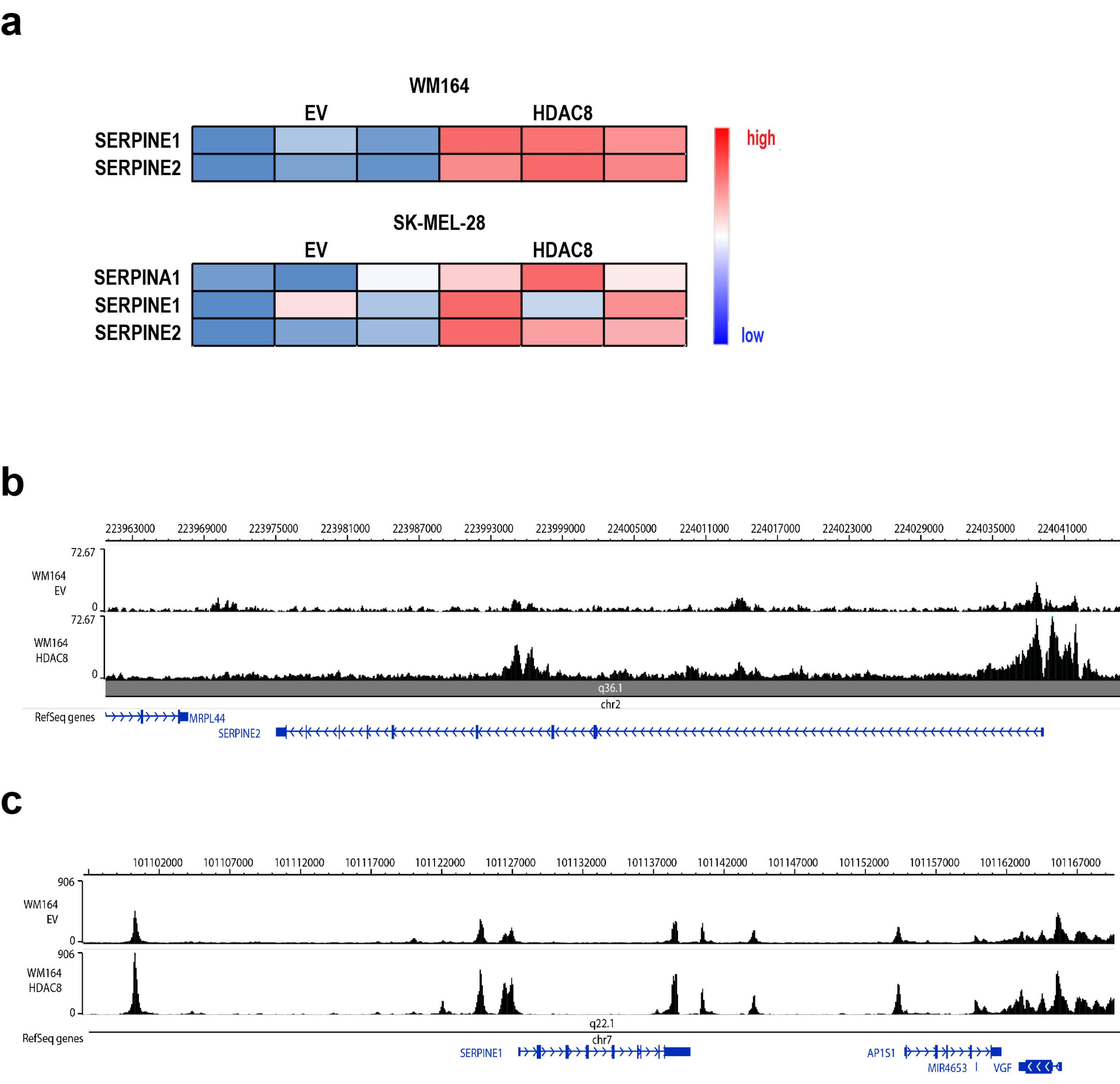

Supplemental Figure 12: HDAC8 upregulates expression of Serpins A1, E1 and E2.
a: Gene expression of SERPINE1 and SERPINE2 in indicated cell lines was determined by RNA-Seq. b: ChIP-Seq was performed on EV and HDAC8 expressing WM164 cells using an acetyl-H3K27 antibody followed by interrogation of gene promoter regions. c: ATAC-Seq was performed on HDAC8 expressing and EV expressing WM164 cells. Regions around SERPINE1's transcriptional start site were investigated for changes in accessibility.

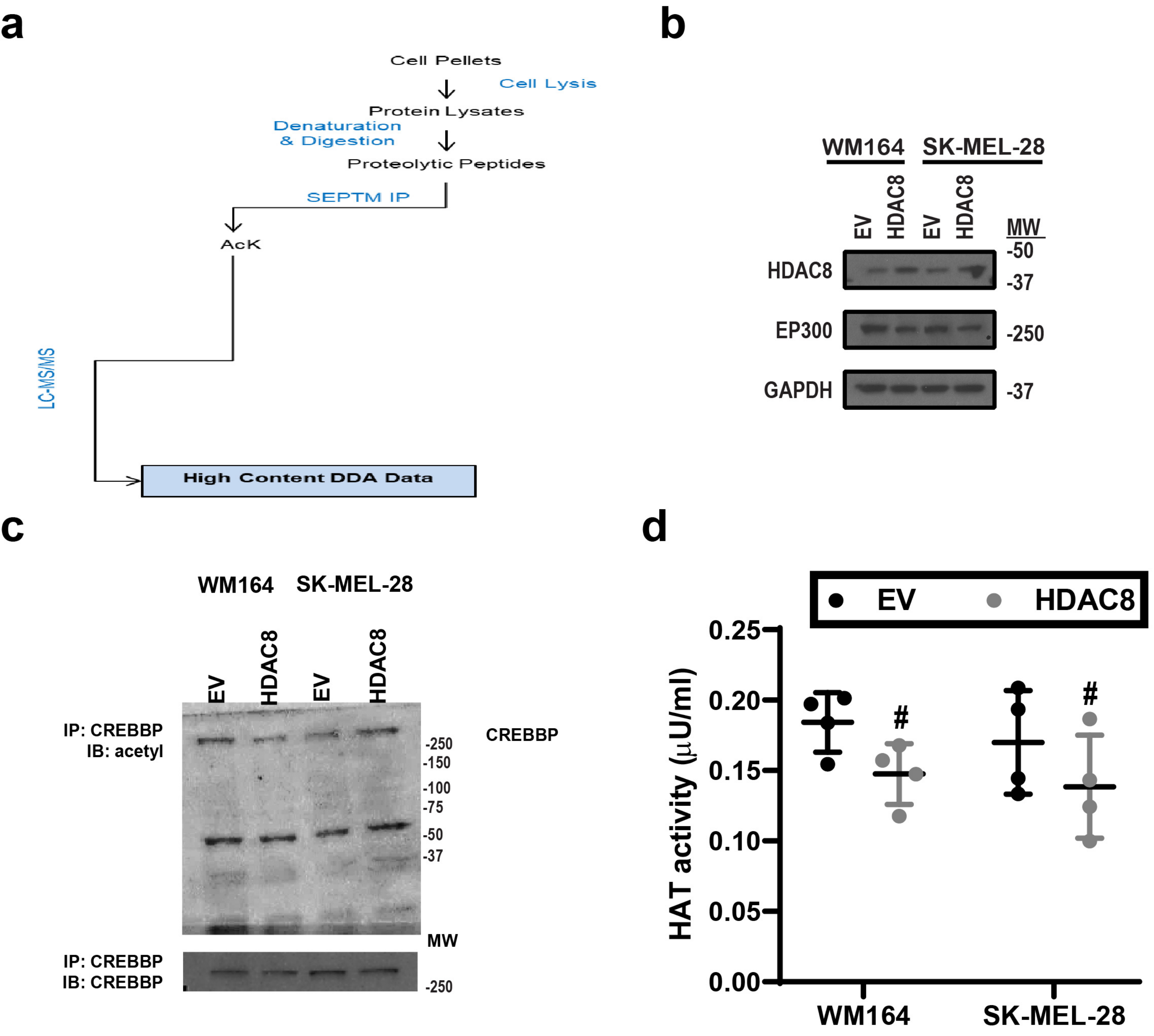

Supplemental Figure 13: HDAC8 expression did not significantly decrease CREBBP HAT activity.
a: Sheared EV and HDAC8 expressing peptide lysates were immunoprecipitated with an anti-acetyl lysine (AcK) antibody. Acetylated peptides were identified using liquid chromatography tandem mass spectrometry (LC-MS/MS) peptide sequencing b: Indicated cell lines were probed for anti-HDAC8 and anti-EP300 antibodies by immunoblot. c: Immunoprecipitation assays were performed with CREBBP in the cell lines indicated. Lysates were probed for acetyl-lysine levels and CREBBP levels by immunoblot. d: Collected lysates were analyzed for CREBBP mediated H4 histone acetylation. Significance was determined by a student's t-test with #=p>0.05. Experiments were run 3 independent times with an n of 4 in (d). All data are presented as mean values +/- SD. Source data are provided as a Source Data file.

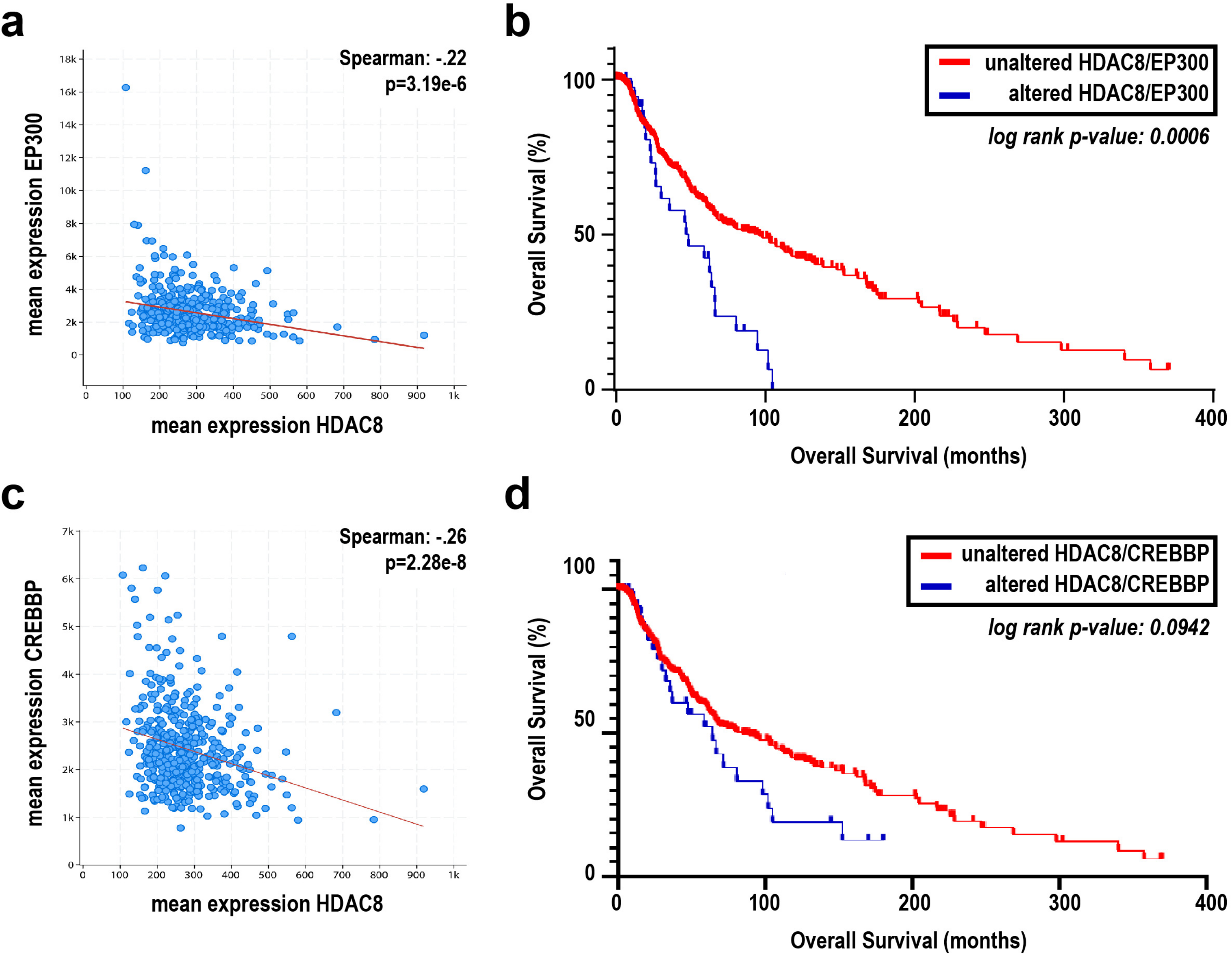

Supplemental Figure 14: Significant alterations in HDAC8/EP300 but not HDAC8/CREBBP mRNA levels conferred a significant decrease in patient survival.

a: Co-expression of HDAC8 and EP300 mRNA was determined using the TCGA PanCancer Atlas melanoma database. Significant negative correlation was determined using a Spearman analysis with significance equaling $p<0.05$. b: Patient samples with decreased CREBBP and increased HDAC8 RNA levels (change in 2-fold expression over all samples) were compared to unaltered patient samples using the TGCA melanoma database. Significance was determined using a log rank test with non-significance equaling $p>0.05$. c: Co-expression of HDAC8 and CREBBP mRNA was determined using the TCGA PanCancer Atlas melanoma database. Significant negative correlation was determined using a Spearman analysis with significance equaling $p<0.05$. d: Patient samples with decreased CREBBP and increased HDAC8 RNA levels (change in 2-fold expression over all samples) were compared to unaltered patient samples using the TGCA melanoma database. Significance was determined using a log rank test with non-significance equaling $p>0.05$.

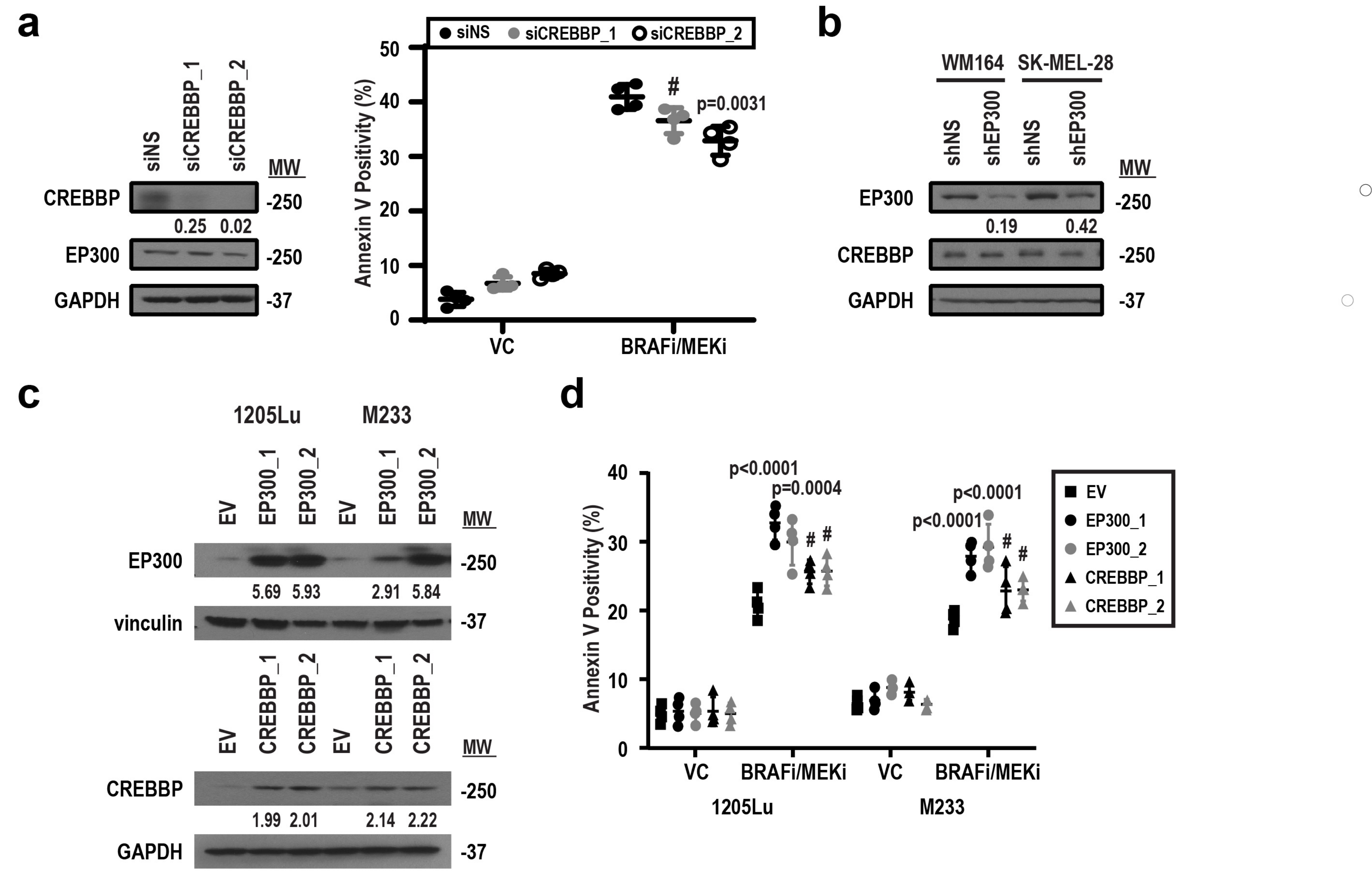

Supplemental figure 15: Overexpressing EP300 increased BRAFi sensitivity in melanoma.

a: WM164 cells were transfected with a non-silencing siRNA (siNS) or an CREBBP siRNA . Cells were probed for protein expression of EP300 and CREBBP after 72 hours transfection. Cells were treated with dabrafenib and trametinib (100 nmol/L dabrafenib, 10 nmol/L trametinib, BRAFi/MEKi) for 72 hours. Apoptosis was measured by Annexin V APC staining using flow cytometry. b: Indicated cell lines were treated with shEP300 or shNS control. Cells were probed for EP300 or CREBBP using a western blot. c: Cells were stably transfected with an empty vector, EP300 or CREBBP overexpressing plasmid. Cells were probed for EP300 and CREBBP by western blot and protein levels of EP300 and CREBBP were quantified by ImageJ. d: Cells were treated with dabrafenib and trametinib (100 nmol/L dabrafenib, 10 nmol/L trametinib, BRAFi-MEKi) for 72 hours. Apoptosis was measured by Annexin V APC staining using flow cytometry. Significance in (a) and (d) was determined by a one-way ANOVA followed by a post hoc t-test with **=p<0.01, *=p<0.05, and #=p>0.05. All experiments were run 3 independent times with an n of 4 in each cohort in. All data are presented as mean values +/- SD. Source data are provided as a Source Data file.

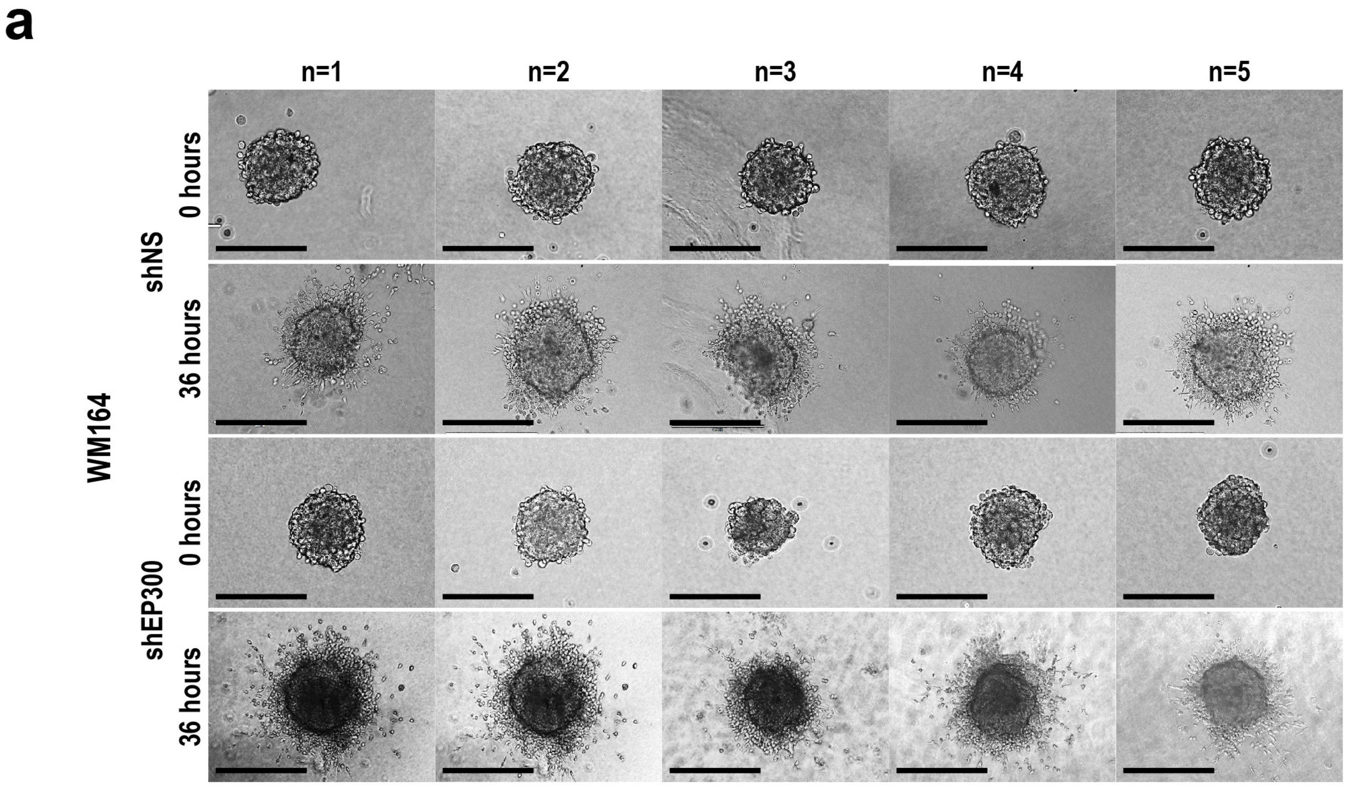

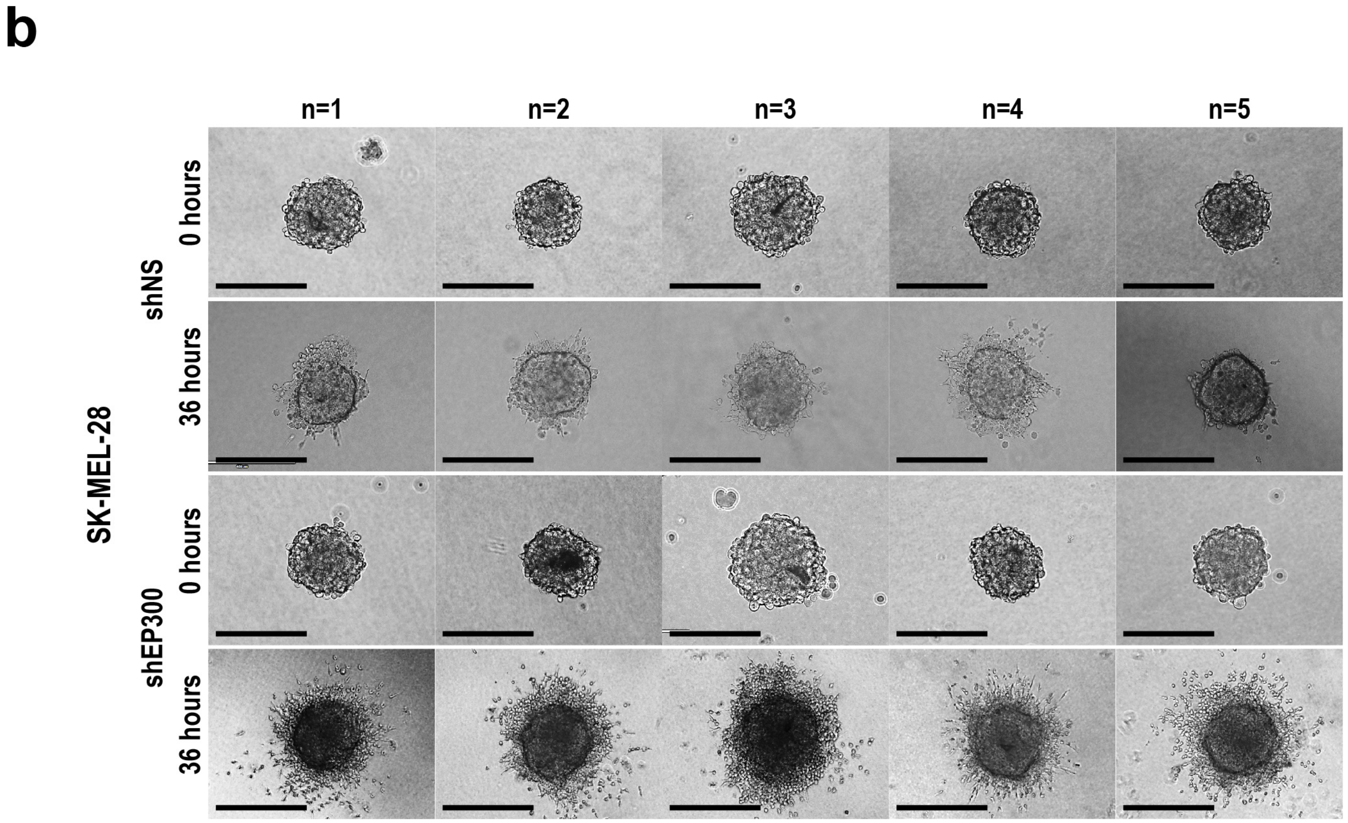

Supplemental figure 16: Knocking down EP300 increases invasion in a 3D collagen assay
a-b: Cells were grown in collagen for 36 hours then imaged using an EVOS 5100 autofluorescence imager. Scale bar = 500 µm. a: Raw images for 3D collagen assay performed in WM164 cells shown in figure 7h.
b: Raw images for 3D collagen assay performed in SK-MEL-28 cells shown in figure 7i.

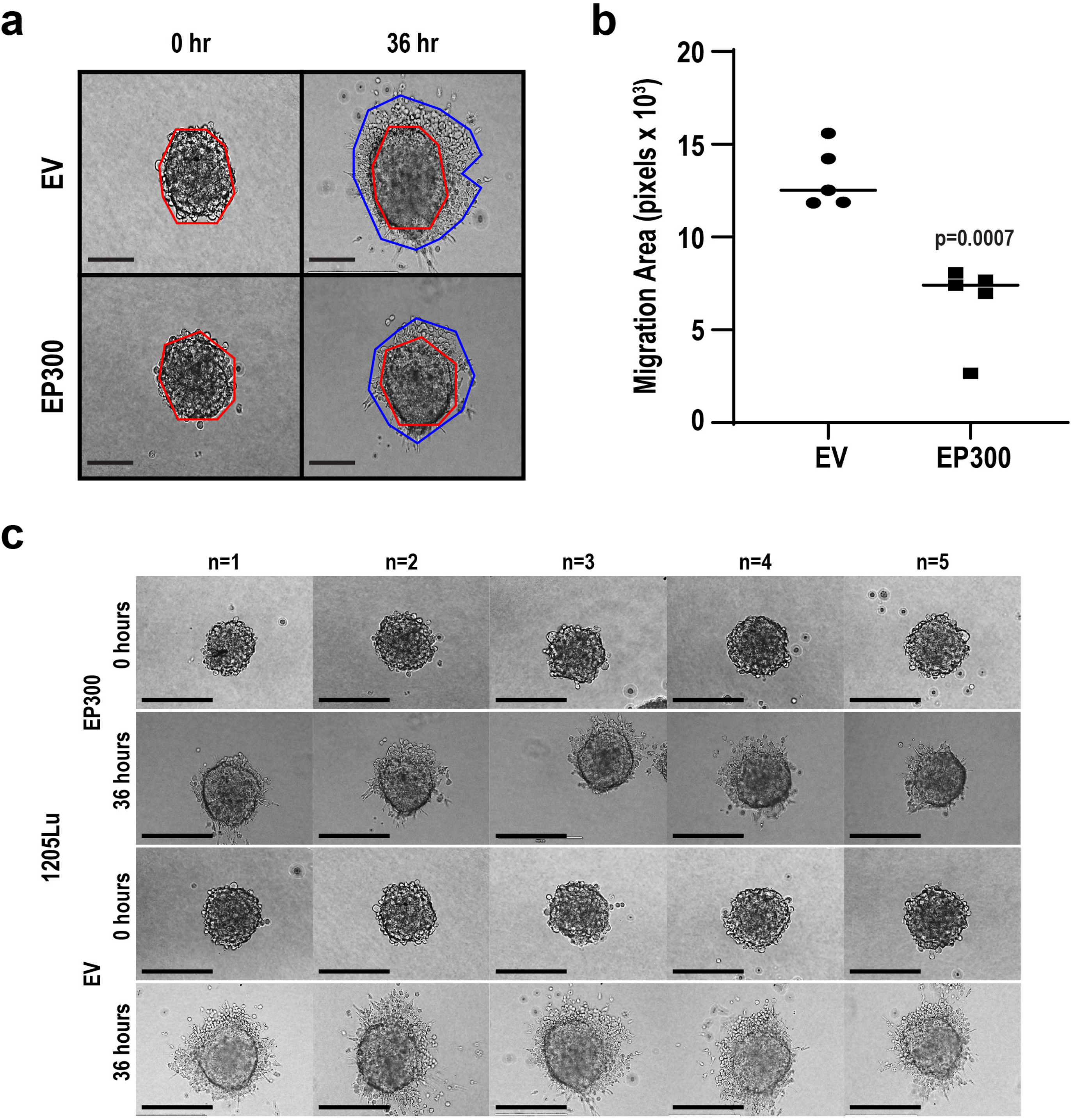

Supplemental figure 17: Overexpressing EP300 decreased migration in melanoma.
a: EP300- or EV-expressing spheroids were plated on collagen for indicated time points. Scale bars = 500 µm. **b:** Spheroid invasion area was quantified using ImageJ software. Significance was determined by students t-test with ***=p<0.001. Experiments were repeaated in triplicate with an n of 5 in each experiment. All data are presented as mean values +/- SD. c. Raw image files are shown for 3D collagen invasion assay. Source data are provided as a Source Data file.

a

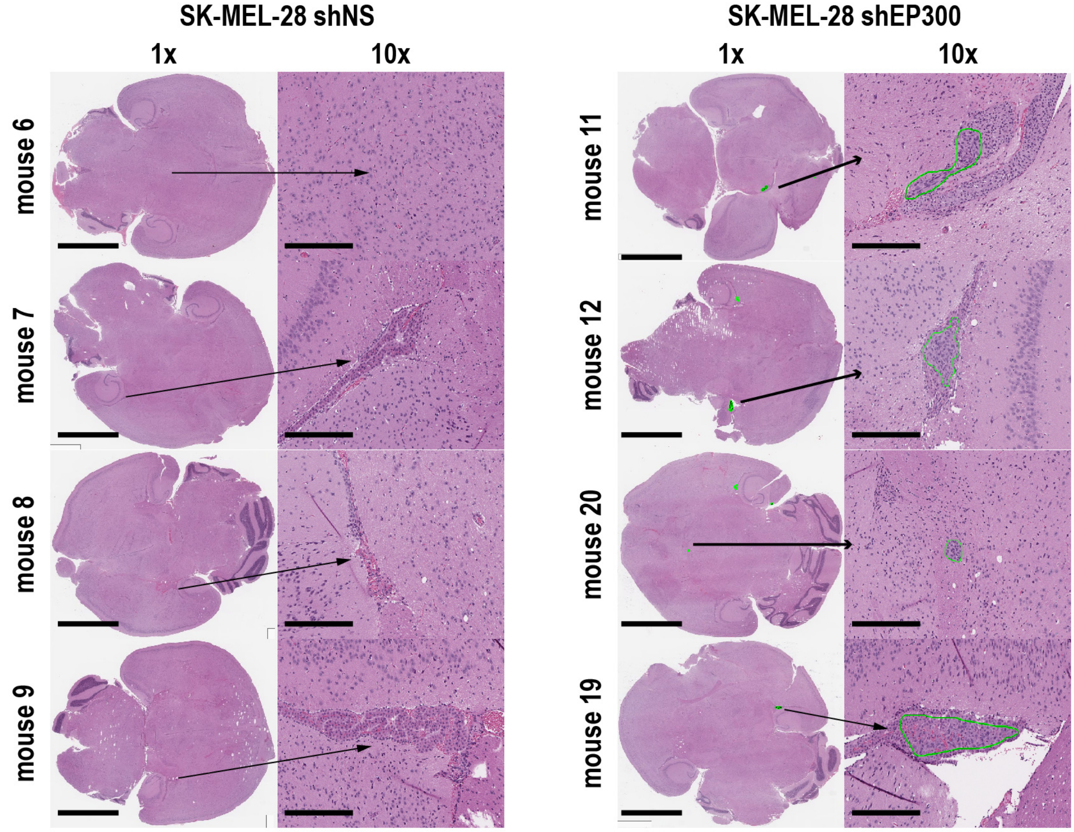

b

shNS

	mouse #	1	2	3	4	5	6	7	8	9
	weight	20.3	21.4	19.8	20.1	20.8	17.4	18.7	22.1	19.5
tumor size (μm^2)	metastases 1	na	na	na	na	na	na	na	na	na
	metastases 2	na	na	na	na	na	na	na	na	na
	metastases 3	na	na	na	na	na	na	na	na	na

shEP300

	mouse #	11	12	13	14	15	16	17	18	19	20
	weight	19.8	18.5	21.3	20.5	19.8	18.9	22.3	19.4	18.7	17.9
tumor size (μm^2)	metastases 1	4022	18188	na	na	na	24057	na	na	15879	5494
	metastases 2	28604	56795	na	na	na	47490	na	na	na	24698
	metastases 3	na	na	na	na	na	na	na	na	na	2668

Supplemental Figure 18: Knocking down EP300 increases invasion to the brain.
a: shNS and shEP300 expressing SK-MEL-28 cells were introduced into NOD.CB17-Prkdcscid/J mice by intracardiac injection. Tumors were allowed to establish for 14 days. Brains were extracted and stained for tumors by H&E. 1x scale bars=4 mm. 10x scale bars=300 μm. b: Number of tumors and tumer sizes for each animal were quantified using Aperio Imagescope. Tumor weights were taken at endpoint.

a

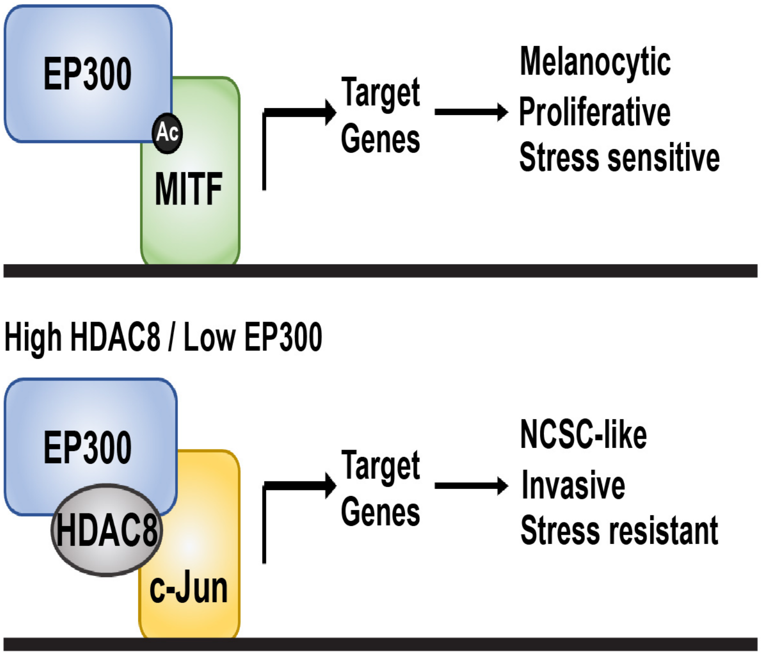

Supplemental figure 19: Proposed mechanism of HDAC8 and EP300 interaction on melanoma phenotype switching.
a: A schematic of the proposed mechanism of action for the HDAC8/EP300 modulated phenotype switch is shown.
