# Supplementary material for: HDAC8-mediated inhibition of EP300 drives a transcriptional state that increases melanoma brain metastasis
Source: Nat Commun. 2023 Nov 29;14:7759. doi: 10.1038/s41467-023-43519-1 (PMC10686983; doi:10.1038/s41467-023-43519-1)
Supplement: Supplementary file 2 — Description of Additional Supplementary Files [file 41467_2023_43519_MOESM2_ESM.pdf]

# <u>Description of Additional Supplementary Files</u>

**Supplementary Data 1:** Genes used to define the 4 melanoma cell states

**Supplementary Data 2:** acetylated peptides identified
