# Supplementary material for: HDAC8-mediated inhibition of EP300 drives a transcriptional state that increases melanoma brain metastasis
Source: Nat Commun. 2023 Nov 29;14:7759. doi: 10.1038/s41467-023-43519-1 (PMC10686983; doi:10.1038/s41467-023-43519-1)
Supplement: Supplementary file 5 — Reporting Summary [file 41467_2023_43519_MOESM5_ESM.pdf]

nature portfolio

Corresponding author(s): Dr. Keiran Smalley

Last updated by author(s): 5.10.2023

Reporting Summary

Nature Portfolio wishes to improve the reproducibility of the work that we publish. This form provides structure for consistency and transparency in reporting. For further information on Nature Portfolio policies, see our Editorial Policies and the Editorial Policy Checklist.

Statistics

For all statistical analyses, confirm that the following items are present in the figure legend, table legend, main text, or Methods section.

n/a	Confirmed	
☐	☒	The exact sample size (*n*) for each experimental group/condition, given as a discrete number and unit of measurement
☐	☒	A statement on whether measurements were taken from distinct samples or whether the same sample was measured repeatedly
☐	☒	The statistical test(s) used AND whether they are one- or two-sided<br>*Only common tests should be described solely by name; describe more complex techniques in the Methods section.*
☐	☒	A description of all covariates tested
☐	☒	A description of any assumptions or corrections, such as tests of normality and adjustment for multiple comparisons
☐	☒	A full description of the statistical parameters including central tendency (e.g. means) or other basic estimates (e.g. regression coefficient) AND variation (e.g. standard deviation) or associated estimates of uncertainty (e.g. confidence intervals)
☐	☒	For null hypothesis testing, the test statistic (e.g. *F*, *t*, *r*) with confidence intervals, effect sizes, degrees of freedom and *P* value noted<br>*Give P values as exact values whenever suitable.*
☒	☐	For Bayesian analysis, information on the choice of priors and Markov chain Monte Carlo settings
☒	☐	For hierarchical and complex designs, identification of the appropriate level for tests and full reporting of outcomes
☐	☒	Estimates of effect sizes (e.g. Cohen's *d*, Pearson's *r*), indicating how they were calculated

Our web collection on statistics for biologists contains articles on many of the points above.

Software and code

Policy information about availability of computer code

Data collection	For RNA-Seq, library preparation and sequencing were performed by Novogene using TruSeq RNA Library Preparation Kit and NovaSeq 6000 (Illumina, San Diego, CA) with PE150. For ChIP-Seq, the libraries were purified, size-selected for between 200 bp and 700 bp using Ampure beads XP (Beckman Coulter, Brea, CA), and pooled for Novaseq 6000 54 2x150 flow cell (ICBR Next-Gen Sequencing Core, University of Florida). For ATAC-Seq, sequencing of pooled libraries was performed on a Novaseq 6000 S4 2x150 flow cell (ICBR Next-Gen Sequencing Core, University of Florida). For ChIP, DNA was run on a 7900HT Fast Real-Time PCR System (Thermo Fisher Scientific) for 40 cycles using TaqMan master mix (Applied Biosystems, Waltham, MA). For acetylomics, a nanoflow ultra-high performance liquid chromatograph (RSLC, Dionex, Sunnyvale, CA) interfaced with an electrospray bench top quadrupole-orbitrap mass spectrometer (Q Exactive Plus, Thermo Fisher Scientific) was used for liquid chromatography tandem mass spectrometry (LC-MS/MS) peptide sequencing experiments.
Data analysis	For RNA-Seq, short reads were filtered and trimmed using Trimmomatic (v 0.36). QC on the original and trimmed reads was performed using FastQC (v 0.11.4) and MultiQC (v 1.1). The reads were aligned to the transcriptome using STAR (v 2.7.3a). Transcript abundance was quantified using RSEM (v 1.2.31). Gene ontology was carried out using GSEA and ShinyGO platforms. For ChIP-Seq, the input sequences were trimmed using Trimmomatic. Quality control was performed before and after trimming using FastQC. The input sequences were then aligned to the GRCh38 genome using Bowtie (v 2.3.5.1). Peak detection was performed using MACS (v 2.1.2) and motif finding on peak regions was performed with the HOMER find-Motifs function. For ATAC-Seq, reads were trimmed using Trimmomatic (v 0.36), and QC on the original and trimmed reads was performed using FastQC (v 0.11.4) and MultiQC (v 1.1). The reads were aligned to the human genome version GRCh38 using Bowtie (v. 2.3.3), and ATAC peak

calling was performed using the MACS (v 2.1.2). Differential peak analysis was performed using DASA (https://github.com/uf-icbr-bioinformatics/dasa). For acetylomics, database searches were performed with Mascot (Matrix Science, Boston, MA) and MaxQuant. Gene ontology of significantly deacetylated proteins were carried out using the STRING protein-protein interaction network. All flow cytometry analyses were analyzed using FloJo software (vl0.7.1). Protein quantification was carrried out using ImageJ. IHC images were analyzed using Aperio Imagescape (v.12.3.3.5048). For all other analyses, Graph pad 8 was used.

For manuscripts utilizing custom algorithms or software that are central to the research but not yet described in published literature, software must be made available to editors and reviewers. We strongly encourage code deposition in a community repository (e.g. GitHub). See the Nature Portfolio guidelines for submitting code & software for further information.

Data

Policy information about availability of data

All manuscripts must include a data availability statement. This statement should provide the following information, where applicable:

- Accession codes, unique identifiers, or web links for publicly available datasets
- A description of any restrictions on data availability
- For clinical datasets or third party data, please ensure that the statement adheres to our policy

The raw sequence reads, raw counts and differential expression for RNA-seq data generated in this study have been deposited in the Gene Expression Omnibus (GEO) [https://www.ncbi.nlm.nih.gov/geo/query/acc.cgi?acc=GSE218625] (GSE218625) and [https://www.ncbi.nlm.nih.gov/geo/query/acc.cgi?acc=GSE240307] (GSE240307]). The raw sequence reads for ATAC-Seq and ChIP-Seq data generated in this study have been deposited with links to BioProject accession number PRJNA903203 in the NCBI BioProject database [https://www.ncbi.nlm.nih.gov/bioproject/?term=prjna903203]. The raw reads for the proteomic data generated in this study have been deposited in the ProteomeXchange Consortium via the PRIDE partner repository with the dataset identifier PXD044471 [https://www.ebi.ac.uk/pride/archive/projects/PXD044471]. The raw data files for the previously published and publicly available scRNA-seq data analyzed in this study have been deposited in the Gene Expression Omnibus (GSE174401) [https://www.ncbi.nlm.nih.gov/geo/query/acc.cgi?acc=GSE174401]. Source data are provided with this paper.

Research involving human participants, their data, or biological material

Policy information about studies with human participants or human data. See also policy information about sex, gender (identity/presentation), and sexual orientation and race, ethnicity and racism.

Reporting on sex and gender	N/A
Reporting on race, ethnicity, or other socially relevant groupings	N/A
Population characteristics	N/A
Recruitment	N/A
Ethics oversight	N/A

Note that full information on the approval of the study protocol must also be provided in the manuscript.

Field-specific reporting

Please select the one below that is the best fit for your research. If you are not sure, read the appropriate sections before making your selection.

[x] Life sciences [ ] Behavioural & social sciences [ ] Ecological, evolutionary & environmental sciences

For a reference copy of the document with all sections, see nature.com/documents/nr-reporting-summary-flat.pdf

Life sciences study design

All studies must disclose on these points even when the disclosure is negative.

Sample size	No statistical analysis was used to predetermine sample size. At least an n of 3 was used each time for reproducibility concerns. For experiments with a smaller sample size, at least 5 n's were collected to strengthen the data. For samples with a larger sample size, at least 3 samples per experiment were used to strengthen the data.
Data exclusions	N/A

Replication

Experimental n's were taken on 3 different days for all in vitro non-sequencing assays. In vivo experiments were done in duplicate in 3 different cell lines to increase replication. All sequencing experiments excluding ChIP-Seq were done in triplicate. ChIP-Seq was performed in multiple cell lines. For individual experiments, n's were taken from different samples, not multiple times for a single sample. For this manuscript, all attempts of replication were successful.

Randomization

For in vivo metastases experiments, mice were randomized into 2 distinct groups based on weight before intracardiac injections to account for fluctuations in weight. For all other experiments, normalization methods, including using the same cell counts and same protein weight, were used so randomization was not needed due to lack of variability between samples.

Blinding

Technicians performing intracardiac experiments were asked to mark one cell line A and the other B
during injections. The key was accessed after analysis of brain IHCs. For these crucial in vivo experiments, investigators were blinded during collection of organs and during analyses of IHC samples. Cell lines were revealed after analysis.

Reporting for specific materials, systems and methods

We require information from authors about some types of materials, experimental systems and methods used in many studies. Here, indicate whether each material, system or method listed is relevant to your study. If you are not sure if a list item applies to your research, read the appropriate section before selecting a response.

Materials & experimental systems

n/a	Involved in the study	
☐	☒	Antibodies
☐	☒	Eukaryotic cell lines
☒	☐	Palaeontology and archaeology
☐	☒	Animals and other organisms
☒	☐	Clinical data
☒	☐	Dual use research of concern
☒	☐	Plants

Methods

n/a	Involved in the study	
☐	☒	ChIP-seq
☐	☒	Flow cytometry
☒	☐	MRI-based neuroimaging

Antibodies

Antibodies used

The anti-HDAC8 antibody was a gift from Dr. Ed Seto and was made by Dr. Seto. Antibodies against SMC3 (# 5696, D47B5), p-EGFR (Y1068, # 3777, D7A5), EGFR (#4267, D38B1), p-c-Jun (S73, #3270, D47G9), c-Jun (# 9165, 60A8), HDACl (#34589, D5C6U), HDAC2 (#57156, D6S5P), HDAC3 (#85057, D2O1K), acetyl-histone 3 (Lys27, #8173, D5E4), histone3 (#4499, D1H2), an anti-acetyl antibody (CST, #9441), EP300 (# 86377, D8Z4E), and CREBBP (# 7389,D6C5) were purchased from Cell Signaling Technologies (Danvers, MA). An anti-acetyl-SMC3 antibody was a kind gift from Forma Therapeutics and formulated in house. An Anti-MITF (NBll0-10872) antibody was ordered from Novus (Littleton, CO). Anti-vinculin (V9131), and-GAPDH (G9545) antibodies were ordered from Millipore Sigma. For histone markers, an anti-HDAC8 (Ab187139) and anti-histone 4(Ab7311) antibody was ordered from Abeam. Beta-tubulin (86298s), Histone-H3 (4499s) and a histone-H4 (2935c) antibody were ordered from Cell Signaling Technology. H3K9ac antibody (61251) was ordered from Active Motif. A H4K16ac antibody (MA5-27794) was ordered from lnvitrogen while a H4K20ac antibody (13-0039) was ordered from EpiCypher. For ChIP-Seq assays, antibodies were used including anti-H3K27ac (Active Motif, #39133, Carlsbad, CA), anti-H3K27me3 (CST, #9733), and IgG (CST, #3900).

Validation

HDAC8 was validated by Dr. Ed Seto in the manuscript "Negative Regulation of Histone Deacetylase 8 Activity by Cyclic AMP-Dependent Protein Kinase A" from 2003. Specifically, the rabbit polyclonal anti-HDAC8 antibody was raised against a GST-tagged HDAC8 fusion protein containing the C-terminal region, residues 305 to 377, of HDAC8. The anti-acetyl-SMC3 antibody has been validated by Forma and previously cited in "HDAC8 Regulates a Stress Response Pathway in Melanoma to Mediate Escape from BRAF Inhibitor Therapy" from 2019.

For commercially available antibodies:

Cell signaling:

SMC3: Rabbit Monoclonal antibody is produced by immunizing animals with a synthetic peptide corresponding to residues surrounding Arg132 of human SMC3 protein. 15 Citations including “Genetic analysis of cancer drivers reveals cohesin and CTCF as suppressors of PD-L1.” from 2022.

p-EGFR: Rabbit Monoclonal antibody is produced by immunizing animals with a synthetic phosphopeptide corresponding to residues surrounding Tyr1068 of human EGF receptor. 925 Citations including “HCK induces macrophage activation to promote renal inflammation and fibrosis via suppression of autophagy.” from 2023

EGFR: Rabbit Monoclonal antibody is produced by immunizing animals with a fusion protein containing the cytoplasmic domain of human EGF receptor. 1229 citaions including “HCK induces macrophage activation to promote renal inflammation and fibrosis via suppression of autophagy.” from 2023

p-c-Jun: Rabbit Monoclonal antibody is produced by immunizing animals with a synthetic phosphopeptide corresponding to residues around Ser73 of human c-Jun. 378 citations including “

Leucine-973 is a crucial residue differentiating insulin and IGF-1 receptor signaling.” from 2023

c-Jun: Rabbit Monoclonal antibody is produced by immunizing animals with a GST-c-Jun protein corresponding to the amino-terminal sequence of human c-Jun. 1032 citations including “Leucine-973 is a crucial residue differentiating insulin and IGF-1 receptor signaling.” from 2023.

HDAC1: Rabbit Monoclonal antibody is produced by immunizing animals with a synthetic peptide corresponding to residues surrounding Ala440 of human HDAC1 protein100 citations including “TRIM5α recruits HDAC1 to p50 and Sp1 and promotes H3K9

deacetylation at the HIV-1 LTR." from 2023.
HDAC2: Rabbit Monoclonal antibody is produced by immunizing animals with a synthetic peptide corresponding to residues surrounding His438 of human HDAC2 protein. 66 citations including "Acute liver steatosis translationally controls the epigenetic regulator MIER1 to promote liver regeneration in a study w..." from 2023.
HDAC3: Rabbit Monoclonal antibody is produced by immunizing animals with a synthetic peptide corresponding to residues surrounding Gly416 of human HDAC3 protein. 41 citations including "Hypoxia induces HIF1α-dependent epigenetic vulnerability in triple negative breast cancer to confer immune effector dysf..." from 2022
histone 3: Rabbit Monoclonal antibody is produced by immunizing animals with a synthetic peptide corresponding to the carboxy terminus of the human histone H3 protein. 1504 citations including "Class 3 PI3K coactivates the circadian clock to promote rhythmic de novo purine synthesis.: from 2023
acetyl histone h3: Acetyl Monoclonal antibody is produced by immunizing animals with a synthetic peptide corresponding to residues surrounding acetylated Lys27 of human histone H3 protein. 379 citations including "Lactate-dependent transcriptional regulation controls mammalian eye morphogenesis." from 2023
acetyl: Rabbit Polyclonal antibodies are produced by immunizing animals with a synthetic acetylated lysine-containing peptide. Antibodies are purified by protein A and peptide affinity chromatography. 941 citations including "Chemoproteomic target deconvolution reveals Histone Deacetylases as targets of (R)-lipoic acid." from 2023
EP300: Rabbit Monoclonal antibody is produced by immunizing animals with a synthetic peptide corresponding to residues near the carboxy terminus of human p300 protein. 45 citations including "METTL3 acetylation impedes cancer metastasis via fine-tuning its nuclear and cytosolic functions." from 2022
CREBBP: Rabbit Monoclonal antibody is produced by immunizing animals with a recombinant protein specific to the amino terminus of human CBP protein. The epitope has been mapped to residues surrounding Ser235. 121 citations including "Osteocytes directly regulate osteolysis via MYD88 signaling in bacterial bone infection." from 2022
Beta-tubulin: Mouse Monoclonal antibody is produced by immunizing animals with a synthetic peptide corresponding to the amino terminus of human β-tubulin protein. 142 citations including "Resilience to autosomal dominant Alzheimer's disease in a Reelin-COLBOS heterozygous man." from 2023
histone 4: Mouse Monoclonal antibody is produced by immunizing animals with a synthetic peptide corresponding to the amino-terminal sequence of human histone H4. 84 citations including "Dynamic antagonism between key repressive pathways maintains the placental epigenome." from 2023
IgG control: Rabbit (DA1E) mAb IgG XP® Isotype Control is not directed against any known antigen. It functions as an isotype control for rabbit IgG antibodies. 641 citations including "Spatially resolved multiomics of human cardiac niches." from 2023
Novus:
MITF: Mouse monoclonal antibody against N-terminal fragment of human microphthalmia protein. citations include "Xu Z, Li Y, Wang D et al. Mutated SASH1 promotes Mitf expression in a heterozygous mutated SASH1 knock in mouse model Int. J. Mol. Med. 2020-06-19 [PMID: 32582980] (WB, IF/IHC, Mouse)"
Millipore Sigma:
vinculin: Monoclonal Anti-Vinculin (mouse IgG1 isotype) is derived from the hVIN-1 hybridoma produced by the fusion of mouse myeloma cells and splenocytes from immunized BALB/c mice. Citations include "Vinculin and talin: focus on the myocardium" from 2009.
GAPDH: Synthetic peptide corresponding to amino acids of mouse GAPDH, conjugated to KLH via an N-terminal cysteine residue. Cited in "Integrated stress response modulates cellular redox state via induction of cystathionine γ-lyase: cross-talk between integrated stress response and thiol metabolism." from 2012
HDAC8:30 citations including " Kim JY et al. HDAC8-Selective Inhibition by PCI-34051 Enhances the Anticancer Effects of ACY-241 in Ovarian Cancer Cells. Int J Mol Sci 23:N/A (2022)."
active motif:
H3K9ac: The Histone H3 acetyl Lys9 antibody was raised against a peptide including acetyl-lysine 9 of human Histone H3. NGS-QC® certification: this antibody has been processed by the NGS-QC® generator.
H3K27ac: NGS-QC® certification: this antibody has been processed by the NGS-QC® generator. This Histone H3 acetyl Lys27 antibody was raised against a peptide including acetyl-lysine 27 of histone H3.
Invitrogen:
H4K16ac: Carrier-protein conjugated synthetic peptide encompassing a sequence within the N-terminus region of human Histone H4 (acetyl Lys16). The exact sequence is proprietary.

Eukaryotic cell lines

Policy information about cell lines and Sex and Gender in Research

Cell line source(s)	The FOM173, NHEM, 1205Lu, A375, M233, SK-MEL-28, WM164, WM793, and WM983A cell lines were a generous gift from Dr. Meenhard Herlyn (The Wistar Institute, Philadelphia, PA). HERMES1 and HERMES3 cell lines were acquired from Dr. Dorothy Bennett (St. Georges Hospital Medical School, UK). Human umbilical vein endothelial cells (HUVEC, # PCS-100-013) were acquired from ATCC (Manassas, Virginia).
Authentication	Cell lines were authenticated by ATCC's Human STR human cell line authentication service and cell lines were replaced from frozen stocks after 10 passages. HUVEC cells were discarded after 3 passages.
Mycoplasma contamination	Every 3 months, cells were tested for Mycoplasma contamination using the Plasmotest-Mycoplasma Detection Test (InvivoGen) with the last test date on 06/20/2023 with all cell lines testing negative.
Commonly misidentified lines (See ICLAC register)	No commonly misidentified cell lines were used in this study.

Animals and other research organisms

Policy information about studies involving animals; ARRIVE guidelines recommended for reporting animal research, and Sex and Gender in Research

Laboratory animals	10 week old NOD.CB17-Prkdcscid/J female mice were used in the study. Mice were maintained in a 12 hour light/12 hour dark facility with 65-75 degrees temperature and 40-60% humidity. In all in vivo experiments, a tumor burden resulting in less than 20% animal weight loss for 72 hours, tumor volume below 1500 mm^3, and tumor size below 2 cm was permitted. The maximum tumor burden was not exceeded.
Wild animals	N/A
Reporting on sex	Female mice were used in this study. Sex was not a prerequisite of study design and only used based on availability of animals from provider.
Field-collected samples	N/A
Ethics oversight	The University of South Florida Institutional Animal Care and Use Committee (IACUC) oversees and approves animal studies to ensure that they are consistent with the recommendations of the Guide for the Care and Use of Laboratory Animals, the Animal Welfare Act, the Public Health Service Policy on Humane Care and Use of Laboratory Animals, and the IACUC Principles and Procedures. The university program and facilities for animal care and use are fully accredited by the Association for Assessment and Accreditation of Laboratory Animal Care International (AAALAC). In all in vivo experiments, a tumor burden resulting in less than 20% animal weight loss for 72 hours, tumor volume below 1500 mm^3, and tumor size below 2 cm was permitted. The maximum tumor burden was not exceeded.

Note that full information on the approval of the study protocol must also be provided in the manuscript.

Plants

Seed stocks	N/A
Novel plant genotypes	N/A
Authentication	N/A

ChIP-seq

Data deposition

- [x] Confirm that both raw and final processed data have been deposited in a public database such as GEO.
- [x] Confirm that you have deposited or provided access to graph files (e.g. BED files) for the called peaks.

Data access links<br>*May remain private before publication.*	ChIP-Seq data generated in this study have been deposited with links to BioProject accession number PRJNA903203 in the NCBI BioProject database [https://www.ncbi.nlm.nih.gov/bioproject/]
Files in database submission	WM164 EV numbers 1-3, WM164 HDAC8 numbers 1-3, 1205Lu EV numbers 1-3, 1205Lu HDAC8 numbers 1-3
Genome browser session<br>(e.g. UCSC)	http://genome.ucsc.edu/s/ariva/HDAC8_phenotype_switch

Methodology

Replicates	ChIP-Seq was performed in an n of 1 for 2 separate cell lines consisting of 2 conditions.
Sequencing depth	All reads were 2x150, ie 150bp paired-end. WM164_H3K27ac had 40,579,634 total reads and 33,430,190 mapped reads. WM164_HDAC8_H3K27ac had 42,691,276 total reads and 35,142,970 mapped reads. 1205_H3K27ac had 30,075,164 total reads and 24,873,438 mapped reads. 1205_HDAC8_H3K27ac had 24,678,859 total reads and 19,185,529 mapped reads.
Antibodies	DNA were immunoprecipitated using antibodies including anti-H3K27ac (Active Motif, #39133, Carlsbad, CA) and IgG (CST, #3900) overnight at 4°C.
Peak calling parameters	Read mapping was performed with botwie2 using default parameters. Peak calling was performed with MACS version 2.2.7.1 with

	the following parameters: "broad=N, model=Y, paired=Y, qvalue=0.05". The ChIP file is the BAM file generated by mapping the reads to the genome.
Data quality	All peaks we report are at FDR<=5%. Peaks were not filtered by fold enrichment. Instead, fraction of reads in peaks FRIP is utilized which ideally should be above 20%. In our case: WM164-H3K27ac = 21.3%, WM164-HDAC8-H3K27ac = 23.7%, 1205Lu-H3K27ac=24.6 % and 1205Lu-HDAC8-H3K27ac=23.6%.
Software	For ChIP-Seq, the input sequences were trimmed using Trimmomatic. Quality control was performed before and after trimming using FastQC. The input sequences were then aligned to the GRCh38 genome using Bowtie (v 2.3.5.1). Peak detection was performed using MACS (v 2.1.2)

Flow Cytometry

Plots

Confirm that:

- [x] The axis labels state the marker and fluorochrome used (e.g. CD4-FITC).
- [x] The axis scales are clearly visible. Include numbers along axes only for bottom left plot of group (a 'group' is an analysis of identical markers).
- [x] All plots are contour plots with outliers or pseudocolor plots.
- [x] A numerical value for number of cells or percentage (with statistics) is provided.

Methodology

Sample preparation	Melanoma cell lines were treated with drug for 72 hours, collected and stained with Annexin V APC (BD biosciences, Franklin Lakes, NJ)
Instrument	Fluorescence was read on a BD FACSCanto (BD biosciences).
Software	Apoptotic cells were measured for Annexin V positive populations using FloJo software (v10.7.1).
Cell population abundance	10,000 cells were measured per n.
Gating strategy	Apoptotic cells were measured for Annexin V positive populations.

- [x] Tick this box to confirm that a figure exemplifying the gating strategy is provided in the Supplementary Information.
